# Functional architecture of pancreatic islets identifies a population of first responder cells that drive the first-phase calcium response

**DOI:** 10.1371/journal.pbio.3001761

**Published:** 2022-09-13

**Authors:** Vira Kravets, JaeAnn M. Dwulet, Wolfgang E. Schleicher, David J. Hodson, Anna M. Davis, Laura Pyle, Robert A. Piscopio, Maura Sticco-Ivins, Richard K. P. Benninger

**Affiliations:** 1 Department of Bioengineering, University of Colorado, Anschutz Medical Campus, Aurora, Colorado, United States of America; 2 Barbara Davis Center for Childhood Diabetes, University of Colorado, Anschutz Medical Campus, Aurora, Colorado, United States of America; 3 Institute of Metabolism and Systems Research, University of Birmingham, and Centre for Endocrinology, Diabetes and Metabolism, Birmingham Health Partners, Birmingham, United Kingdom; 4 Department of Pediatrics, University of Colorado School of Medicine, Department of Biostatistics and Informatics, Colorado School of Public Health, Aurora, Colorado, United States of America; University of Southern Denmark, Odense, Denmark, DENMARK

## Abstract

Insulin-secreting β-cells are functionally heterogeneous. Whether there exist cells driving the first-phase calcium response in individual islets, has not been examined. We examine “first responder” cells, defined by the earliest [Ca^2+^] response during first-phase [Ca^2+^] elevation, distinct from previously identified “hub” and “leader” cells. We used islets isolated from Mip-Cre^ER^; Rosa-Stop-Lox-Stop-GCamP6s mice (β-GCamP6s) that show β-cell-specific GCamP6s expression following tamoxifen-induced CreER-mediated recombination. First responder cells showed characteristics of high membrane excitability and lower electrical coupling to their neighbors. The first-phase response time of β-cells in the islet was spatially organized, dependent on the cell’s distance to the first responder cell, and consistent over time up to approximately 24 h. When first responder cells were laser ablated, the first-phase [Ca^2+^] was slowed down, diminished, and discoordinated compared to random cell ablation. Cells that were next earliest to respond often took over the role of the first responder upon ablation. In summary, we discover and characterize a distinct first responder β-cell state, critical for the islet first-phase response to glucose.

## Abstract 2

A hallmark of the early stages in diabetes is the disruption of the first-phase peak of insulin secretion. This study reveals a state of beta cells that drives the first-phase calcium response (a precursor to insulin secretion), challenging the existing paradigm that first-phase calcium response to glucose is not organized within the islet.

## Introduction

Diabetes mellitus is a disease characterized by high blood glucose, caused by insufficient secretion of insulin relative to insulin requirements. β-cells within pancreatic islets of Langerhans secrete insulin and are compromised in diabetes. Early work showed that in mechanically dispersed islets, single β-cells are heterogeneous in the level of insulin release [1]. More recent studies have discovered markers that separate β-cells into distinct populations with differing functional properties. This includes markers that subdivides proliferative-competent β-cells from mature β-cells ([2]; subdivides β-cells with different levels of insulin gene expression, granularity, and secretion [3]; or subdivides β-cells that have differing responsiveness to insulin secretagogues [4]). Furthermore, single-cell high-throughput approaches such as single-cell RNA sequencing (scRNAseq) or mass cytometry separate distinct β-cell populations [5,6]. However, the role of the heterogeneity in the function of the islet is poorly understood.

β-cells are excitable and show elevated electrical activity in response to glucose. Following metabolism of glucose, ATP-sensitive potassium channels (K_ATP_) close, depolarizing the membrane. This membrane depolarization opens voltage-gated calcium channels, elevating intracellular free Ca^2+^ activity ([Ca^2+^]) and triggering insulin release. β-cells are electrically coupled to neighboring β-cells via Connexin36 (Cx36) gap junction channels [7–11], which enables the direct exchange of cations between β-cells [12]. Under low glucose conditions, gap junction channels transmit hyperpolarizing currents that suppress islet electrical activity and insulin release [13–15]. Under elevated glucose gap junction channels coordinate the oscillatory dynamics of islet electrical activity, thereby enhancing first-phase insulin and pulsatile second-phase insulin and glucose tolerance [11,16].

Gap junction coupling is non-uniform throughout the islet [17]. β-cells are also heterogeneous in glucose metabolism and excitability [18]. As a result, at low glucose some β-cells are suppressed more than others by hyperpolarizing currents transmitted from neighboring cells [19]. Conversely at elevated glucose, some β-cells are recruited and/or coordinated more than others by depolarizing currents transmitted from neighboring cells. As such, the response of each β-cell within the islet to glucose is different, reflecting both its intrinsic heterogeneity and its context within the islet. Several studies have sought to identify and characterize functional β-cell states within the islet based on the [Ca^2+^] response under glucose stimulation, together with the use of optogenetic-based constructs and laser ablation. For example, β-cells that show significantly increased connectivity, termed “hubs” or “hub cells” [20,21], disproportionately suppressed islet [Ca^2+^] following targeted hyperpolarization via optogenetic stimulation. Conversely, a population of β-cells disproportionately activated islet [Ca^2+^] following targeted depolarization via optogenetic stimulation [22]. Furthermore, cells that show [Ca^2+^] oscillations that precede the rest of the islet, termed “leader” cells or “wave-origin” [22–24], have also been suggested to drive the oscillatory dynamics of [Ca^2+^].

Both in rodents and in humans, first and second phases of insulin secretion have been distinguished [25,26]. Glucose-stimulated [Ca^2+^] influx into the cell is necessary for both first and second phases of insulin secretion [27]. β-cell [Ca^2+^] is also bi-phasic [28] and is correlated with insulin secretion dynamics [29]. The first-phase of [Ca^2+^] response following low-to-high glucose is transitional, where cells with intrinsic differences in metabolic activity or other properties may be responding differently. The second-phase [Ca^2+^] response at high glucose is steady-state where the majority of cells are equally likely to fire [14], but with [Ca^2+^] oscillations showing differing amplitudes, temporal delay (phase lag), and oscillation frequency [30,31]. While different β-cell subpopulations have been examined during this second-phase [Ca^2+^] response, the role of functional β-cell states during the first-phase [Ca^2+^] has not been examined.

Here, we identify functional β-cell state based on cell dynamics during the first-phase [Ca^2+^] response to glucose and test whether they disproportionately affect islet function. We address what mechanisms underlie their properties and disproportionate effect on islet function and ask whether they overlap with other identified β-cell states.

## Results

### First responder cells are distinct from β-cell functional states associated with second-phase [Ca^2+^]

**Fig 1:**
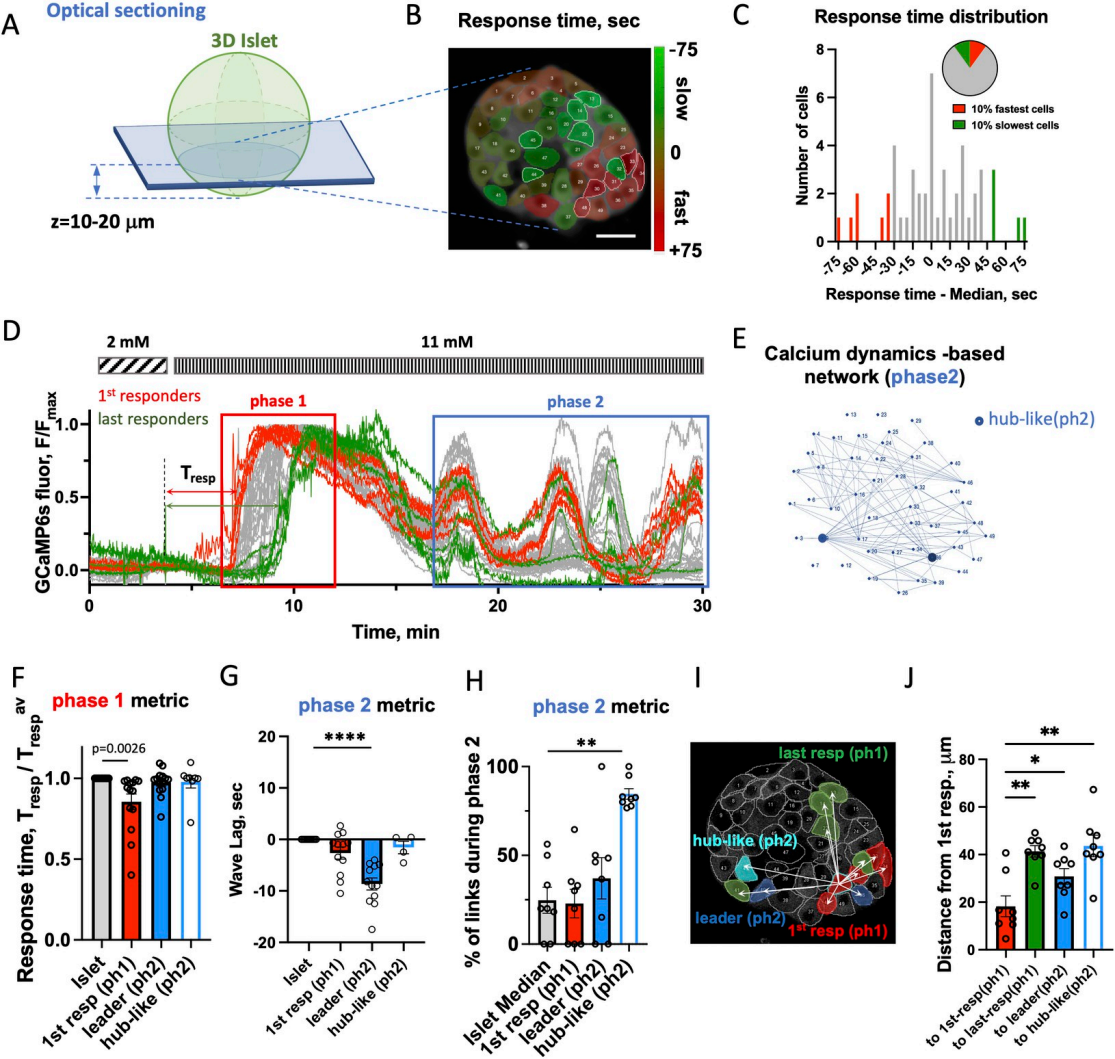
Identification of first responder cells. (**A**) Schematic of where data was collected. (**B**) Representative image of 2D islet plane with false-color map of response time to glucose elevation. (**C**) Representative distribution of response time, Tresp, for the cells shown in (B). Pie chart describes definition of 1st (last) responder cells as 10% of all cells in the islet 2D plane that have fastest (slowest) Tresp. See source data. (**D**) Representative time course of [Ca^2+^] dynamics within an islet shown in (B) following glucose elevation from 2 to 11 mM. In red rectangle: first phase of the [Ca^2+^] response, in blue–second phase. Red curves correspond to first responder (1st-resp) cells and green–to last responder (last-resp) cells. (**E**) Link map based on the functional network analysis performed over the second-phase dynamics in the islet shown in (B and E). Hub-like (ph2) cells are shown as blue dots. (**F**) Phase1 [Ca^2+^] dynamics metric: response time following glucose elevation. Comparison of β-cells leading phase1 (1st responder ph1) vs. phase2 (leader ph2), vs. hub-like (ph2) cells collected from 271 cells, 8–12 islets, 6–7 mice. (**G**) Phase2 [Ca^2+^] dynamics metric: lag of the cell’s [Ca^2+^] wave with respect to the islet-average wave. Comparison of β-cells leading phase1 vs. phase2 vs. hub-like (ph2) cells (*n* = 4–12 islets from 4–7 mice). (**H**) Percent of functional links normalized to max number of links, identified during second phase of [Ca^2+^] dynamics (*n* = 8 islets from 6 mice). (**I**) Example of measurement of the distance from one of the 5 first responder cells in this islet to other cells. (**J**) Quantification for multiple islets (*n* = 8 islets from 6 mice), where each dot represents average distance from all first responder cells to other cell states per islet. Statistical analysis in F, G utilized linear mixed effect model fit by REML with islet and mouse as a random effect (S2 Statistical analysis LMEM), H, J: utilized 1-way ANOVA (with Dunnett’s multiple comparison test), where **** represents *p* < 0.0001, *** *p* < 0.0002, ** *p* < 0.0021, * *p* < 0.0332 for comparison of the islet-average value vs. all other groups. Functional network analysis was performed via binarization and co-activity matrix analysis, as described [20]. See S1 Data file for values used in each graph. LMEM, linear mixed-effects model.

We first sought to identify β**-**cells that may exert control over the first-phase [Ca^2+^] response and test whether they differ from cells reported to control the second-phase [Ca^2+^] response. [Ca^2+^] dynamics were recorded using confocal microscopy in the 2D islet plane (Fig 1A) continuously before and after glucose stimulation in islets isolated from Mip-Cre^ER^; Rosa-Stop-Lox-Stop-GCamP6s mice (β-GCamP6s) that show β-cell-specific GCamP6s expression following tamoxifen-induced CreER-mediated recombination. We quantified the response time to glucose for each cell using initial [Ca^2+^] elevation (Fig 1B) and defined the “first responder” cells as the 10% of cells in the islet with fastest response to glucose elevation, *T*_*resp*_ (Fig 1C and Materials and methods). The “last responder” cells were defined as 10% of islet cells with latest response. The *T*_*resp*_ distribution varied between the islets. The fastest 25% percentile preceded the islet-median response by approximately 10 s, whereas the slowest 25% percentile lagged the islet median by about the same amount of time (see cumulative *T*_*resp*_ distribution for multiple islets in S1A Fig). First responder cells were located closer to the islet periphery (S1B Fig). Islets from the β-GCamP6s mice demonstrated a bi-phasic [Ca^2+^] response to glucose elevation, with the first phase characterized by a steep [Ca^2+^] rise and subsequent plateau (Fig 1D red rectangle), and the second, oscillatory phase characterized by [Ca^2+^] waves (Fig 1D, blue rectangle). Some islets did not have a robust [Ca^2+^] wave (see Materials and methods).

We next examined the overlap between properties of cells defined by the first-phase and second-phase [Ca^2+^] dynamics. During the oscillatory second phase, highly connected “hub cells” have been implicated in maintaining [Ca^2+^] elevations. To assess whether first responder cells overlap with these “hub” cells, we identified “hub-like (phase2)” cells via network analysis as performed previously [20] (Fig 1E). “Leader” cells, which have previously been defined and characterized [22,24], were defined as 10% of all cells with highest negative phase lag, which corresponds to showing an earlier [Ca^2+^] oscillation (S1C and S1D Fig).

The response times of leader and hub-like (ph2) cells were no different from the islet average (Fig 1F), i.e., they did not lead the first-phase [Ca^2+^] response to glucose. While the first responder cells had significantly different *T*_*resp*_ compared to the islet-average cell (*p* = 0.0105). During the second-phase [Ca^2+^] dynamics, the phase lag of the [Ca^2+^] wave of the hub-like (ph2) cells was no different from the islet average (Fig 1G), i.e., they did not lead the second-phase [Ca^2+^] response to glucose. Interestingly, the first responder and leader cells had phase lag that was significantly different from that of islet average (*p* = 0.0476 and *p* < 0.0001 correspondingly), indicating that they both lead second-phase [Ca^2+^] dynamics. However, further analysis showed significant difference between these 2 cell states: leader cells had much earlier calcium wave (greater negative phase lag) compared to the first responder cells (*p* = 0.0001953 determined by LMEM). Thus, based on the first- and second-phase [Ca^2+^] dynamic metrics, first responder cells were distinct from the leader cells.

Comparing number of functional links derived from the network analysis of the [Ca^2+^] wave during the second-phase [Ca^2+^] response to glucose, we found that first responder and leader cells were not different from the islet average (Fig 1H), unlike hub-like (ph2) cells that showed a significantly higher number of links compared to the islet average (*p* = 0.0018). Thus, first responder cells are distinct from previously defined hub cells. Extended analysis of the features of the [Ca^2+^] dynamics (including network analysis revealing hub-like cells in the first-phase [Ca^2+^]) is presented in S1E and S1K Fig.

The range of response times of the cells (the time it takes for the first to last cell to respond) varied between the islets: an islet with the narrowest range responded over 14 s and an islet with the broadest range responded over 292 s range. The range of wave lags of the slow oscillations for different islets varied between 9 and 85 s, and the range of a number of functional links was between 4 and 20 links.

We next looked at the spatial organization of the first-phase [Ca^2+^] response to glucose. Distances between different β-cells were measured in a z-plane approximately 10 to 20 μm away from the surface of the islet’s beta-cell core. (Fig 1A). Distances were measured from each first responder cell in the islet and then averaged (see Fig 1I and 1J and Materials and methods). First responder cells formed clusters of size <20 μm (Fig 1J, indicated by the red bar). The first responder clusters were significantly spatially separated from leader cells (*p* = 0.0208) and from hub-like (ph2) cells (*p* = 0.0056). On average first responder cells were located <30 μm away from leader cells and 45 μm from hub-like (ph2) cells (Fig 1J). The response time of β-cells in the islet following glucose stimulation, in general, was correlated with their relative proximity to a first responder cell, where cells closer to first responder cells responded earlier (S2A Fig). A linear dependence of the response time versus proximity of the first responder was clear for some islets. For other islets location of the first responder cells was such that multiple response domains with a local first responder in the center were formed (examples are shown in S2B Fig). We defined the speed of the response propagation as the slopes of the distance to first responder versus the time of response (S2A Fig). This time over which the initial response to glucose response propagated across the islet was measured here for the first time, and it varied substantially from islet to islet between 0.16 and 3.3 μm/s. Given different islets had substantially different rates of response to glucose, we subsequently normalized the response time by islet.

### Clusters of first responders are spatially consistent

**Fig 2:**
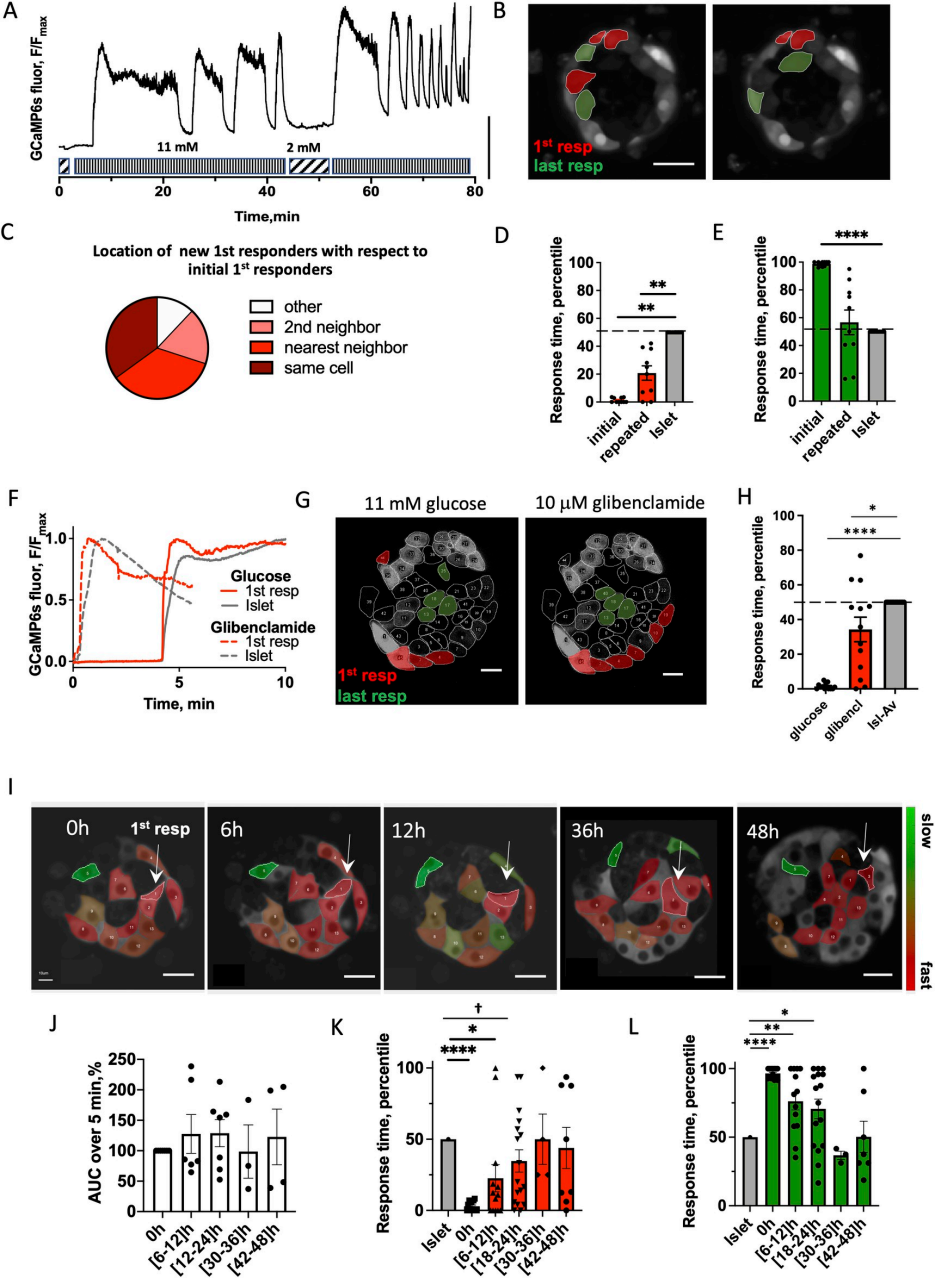
Consistency of first responder cells. (**A**) Representative time course of [Ca^2+^] dynamics within an islet under repeated glucose stimulation. Vertical scale bar represents 30% of the fluorescence intensity change. (**B**) Representative image of the islet with the location of first responders (red) and last responders (green) during initial and repeated glucose elevation. (**C**) Spatial consistency: spatial location of new first responder cells (during repeated glucose elevation) relative to the old first responder cells (during original elevation) (*n* = 10 islets from 4 mice). (**D**) Time of response of cells identified as first responders during the initial glucose elevation and upon repeated glucose elevation (*n* = 10 islets from 4 mice). (**E**) As in (D) for last responders. (**F**) Representative time course of [Ca^2+^] dynamics within an islet under 11 mM glucose stimulation (dashed curve) and under 10 μM glibenclamide (solid curve) stimulation for first responder cell (red) and islet average (gray). (**G**) Representative image of the islet with the location of first responders (red) and last responders (green) during glucose and glibenclamide stimulation. Scale bar indicates 20 μm. (**H**) Time of response of cells identified as first responders during glucose stimulation and upon glibenclamide stimulation (*n* = 13 islets from 7 mice). (**I**) False-color map indicating [Ca^2+^] response time to glucose elevation during the first phase, recorded in the same region of the islet over 48 h at 6 h intervals. Scale bar indicates 20 μm. White arrows point to the 1st responder cell identified at that time. (**J**) AUC for [Ca^2+^] elevation over 0–5 min for the islet average, for each time window indicated. Data are normalized to the [Ca^2+^] AUC at 0 h. (**K**) Time evolution of the response time of the cells identified as first and (**L**) as in (H) for last responders (*n* = 7 islets from 3 mice). Statistical analysis in D, E utilized 1-sample *t* test (with the null hypothesis of initial or repeated difference from the islet being 0). Statistical analysis in H utilized 1-sample *t* test (with the null hypothesis of glucose or glibenclamide difference from the islet being 0). K, L utilized LMEM (S1 Statistical analysis LMEM). † in K indicates *p* = 0.06. See S2 Data file for values used in each graph, and Statistical analysis–Source data for LMEM and 1-sample *t* test details. AUC, area under the curve; LMEM, linear mixed-effects model.

We reasoned that if cells respond randomly to glucose stimulation, then first responder cells identified during the initial glucose elevation will not be the same first responder cells during a repeated glucose elevation. Conversely, if there exists a functional hierarchy of cells within the islet, then there should be consistency in the location of the first responder cells. We first stimulated islets to elevated (11 mM) glucose, lowered back to basal levels, and restimulated over the course of 1 to 2 h (Fig 2A). The location of the first responder cluster in the islet remained consistent between the initial and repeated glucose elevation (Fig 2B and 2C). Some initial first responder cells were first responders in the repeated glucose stimulation (35% of cases), whereas some surrendered their role either to the nearest neighbor cell (35%), or to the 2nd neighbor (18%), or to a more distant cell (12%).

We examined location of the first and last responder cells, as well as leader cells in 3D (S3 Fig). Within 3 cell layers, each separated by 10 μm, the locations of each β-cell subpopulation were conserved, suggesting functional organization in 3D, as we observed in 2D.

### Temporal consistency of first responder cells in not rigid

We next sought to determine whether an early response time was a consistent feature of first responder cells. As above, we first stimulated islets to elevated (11 mM) glucose, lowered back to basal levels, and restimulated over the course of 1 to 2 h (Fig 2A). The period of the observed slow oscillations was in the same range as previously reported [32], (T = 246 ± 89 s). No significant difference was observed in the frequency of the oscillations during the repeated stimulation (0.011 ± 0.008 Hz versus 0.007 ± 0.001 Hz). Response times for all cells during the initial and repeated glucose stimulation are shown in S4 Fig for individual islets. When considering all cells within the islet, there was no significant correlation between the response time of a cell during the initial stimulation compared to the response time upon the repeated stimulation. However, first responder cells in the majority of islets still remained consistent. Last responder cells were not consistent (S4 Fig). We found that temporal consistency of first responder cells (Fig 2D) was substantial, but not fully rigid. Upon restimulation, the initial first responders retained a response that was significantly earlier than the median islet response time: on average in the fastest 25% of the whole *T*_*resp*_ distribution. In contrast, the last-responding cells lacked consistency upon repeated glucose elevation, with a response time close to the islet average (Fig 2E).

We performed sequential glucose and glibenclamide stimulation, in the same manner as repeated glucose stimulation experiments. Glibenclamide is a K_ATP_ channel blocker, resulting in the cell membrane depolarization, mimicking that which happens under glucose-stimulated K_ATP_ channel closure. Following each stimulation, we identified first responder cells (Fig 2F and 2G). Those cells that responded first to glucose, also showed a significantly lower than average response time to glibenclamide (Fig 2G and 2H) (*p* = 0.046). In contrast, those cells that responded last to glucose did not show a response time different to the islet average under glibenclamide. When all cells in the islet plane were considered, we did not observe a significant correlation between the response time during glucose stimulation as compared to the response time upon glibenclamide stimulation. This suggested that while differing K_ATP_ conductance (or resting depolarizing current) may be one factor in defining the earlier response time for a first responder cell, other factors are also likely involved.

### First responder cells represent a cell state rather than a stable subpopulation

To test whether first responder cells are maintained over a longer time, we measured the response time upon elevated glucose within the same cell layer in the islet for 48 h at 6 h intervals. At each time point, we defined the first responder and last responder cells (Fig 2I). There was no significant difference in the total [Ca^2+^] influx in the islet at each time point, suggesting islet remained functional during the culture (Fig 2J). The first responder cells remained consistent during the first 12 h, where they showed significantly earlier [Ca^2+^] response time than the islet average. However, at 18 to 24 h, the first responder cells became less distinguishable from the islet average, and at >24 h, their response time was indistinguishable from the islet average (Fig 2K). A similar temporal pattern was observed for the last responder cells (Fig 2L). Thus, not all β-cells responded randomly to glucose stimulation. Rather, a first responder β-cell state consistently led this response, but this was maintained only over an approximately 24 h time period.

### First responder cells drive first-phase [Ca^2+^] elevation

**Fig 3:**
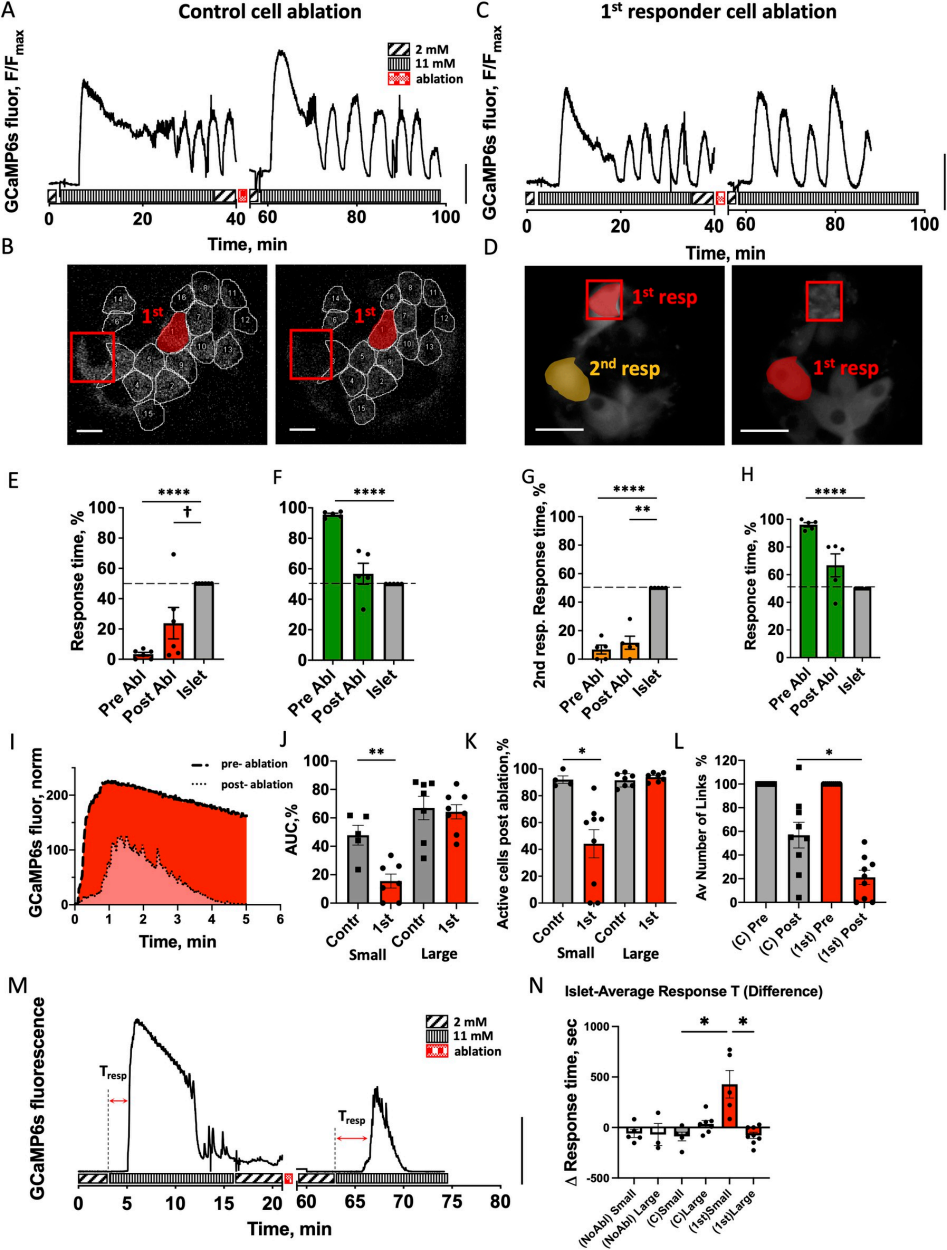
Role of first responder cells. (**A**) Representative time course of [Ca^2+^] dynamics within an islet pre- and post-ablation of a control cell (non-first responder cell). Vertical scale indicates 30% fluorescence intensity change. (**B**) Representative image of the islet with the location of first responders (red) pre- and post-ablation of a control cell (red square). (**C**) As in (A) for ablation of first responder cell. (**D**) As in (B) for ablation of first responder cell (red square), with second responder cell highlighted (orange). (**E**) Time of response of cells identified as first responders prior to control cell ablation and following control cell ablation (*n* = 6 islets from 6 mice). (**F**) As in (E) for last responders. (**G**) Time of response of cells identified as second responders prior to first responder cell ablation and following first responder cell ablation (*n* = 5 islets from 5 mice). (**H**) As in (G) for last responders. (**I**) Representative time course of Ca^2+^ elevation following glucose elevation pre- and post-ablation, with the AUC indicated. (**J**) AUC for the first-phase [Ca^2+^] response post-ablation of a control and first responder cells, for small (<89 μm diameter) and large (>89 μm diameter) islets. Data are normalized to the AUC prior to ablation. (27 islets from 17 mice) (**K**) The % of cells that show [Ca^2+^] elevation post-ablation of a control and first responder cells, for small and large islets. Data are normalized to the AUC prior to ablation. (**L**) Average number of functional links per islet during the first phase of [Ca^2+^] response for a control and first responder cell ablations, for small and large islets combined. Data are normalized to the number of functional links prior to ablation (*n* = 9 islets for each group). Functional network analysis was performed via Pearson-product algorithm, as described before [21]. (**M**) Representative [Ca^2+^] pre- and post-first responder ablation in a small islet, Tresp indicates islet-average response time. (**N**) Response time change in case of no ablation (white bars), control cell ablation (gray bars), or first responder cell ablation (red bars) (35 islets from 20 mice). Two small islets that underwent first responder cell ablation did not respond at all following ablation and were excluded from analysis due to “infinite” response time post-ablation. Statistical analysis in E, F, G, H utilized 1-sample *t* test (with the null hypothesis of pre- or post-difference from the islet being 0), where **** represents *p* < 0.0001, *** *p* < 0.001, ** *p* < 0.01, * *p* < 0.05 indicated for comparison of the groups. † in (E) indicates significance of *p* = 0.06. In I, J, K, L utilized ANOVA with multiple comparison Kruskal–Wallis test, where * *p* < 0.0332 comparing the groups indicated. See S3 Data file for values used in each graph. AUC, area under the curve.

To test whether the hierarchy of cell responsiveness is functionally important, and specifically whether the earliest responding cells disproportionally drive the islet [Ca^2+^] response, we removed single β-cells from the islet via 2-photon-induced femtosecond laser ablation. Two-photon laser ablation allows for highly targeted removal of a cell without disrupting cells in close proximity [33]. As previously, we measured [Ca^2+^] dynamics upon elevation from 2 mM to 11 mM glucose; lowered glucose back to 2 mM, ablated 1 cell, and then repeated glucose elevation (Fig 3A). Under each glucose elevation, we identified first responder cells (Fig 3B). Upon ablation of a control (non-first responder) cell, the islet [Ca^2+^] response was relatively unchanged, with robust second-phase [Ca^2+^] oscillations (Fig 3A and 3B). Upon ablation of a first responder cell, robust second-phase [Ca^2+^] oscillations were also observed (Fig 3C). And a new cell within the islet becomes the first responder cell (Fig 3D).

We examined the [Ca^2+^] response timing and [Ca^2+^] elevation in cells across the islet during the first-phase [Ca^2+^] response to glucose following ablation of either control (non-first responder) cell or first responder cell. Following ablation of the control cell, the response time of first responder cells remained below the islet average (Fig 3E, *p* = 0.0022), whereas last responder cells were not different from the islet average (Fig 3F). On average 53% of the original first responder cells remained as the earliest responding cells (S5A Fig, center pie chart). These findings are very similar to those in the absence of any cell ablation (S5A Fig, left pie chart). Following ablation of a first responder cell, the response time of the next earliest responder cells remained below the islet average (Fig 3H, *p* = 0.0019), whereas last responder cells again were not different from the islet average (Fig 3I). On average 40% of the original next earliest responding cells (“second responder” cells) become new earliest responding cells (S5A Fig, right) and in another 20% of cases they remained as second responder cells. This suggests a hierarchy in the timing and β-cell responsiveness to glucose.

We next quantified the effect of a cell ablation on the whole islet first-phase [Ca^2+^] response. We first defined the [Ca^2+^] influx as the area under the curve (AUC) for [Ca^2+^] over 5 min following elevation (Fig 3K). We observed a size-dependence in AUC in both control and first responder ablation cell experiments (S5B Fig); hence, we separated islets into small and large islets based on the median of the size distribution (S5C Fig). In large islets (>89 μm diameter), the islet-average [Ca^2+^] influx was not different between control or first responder cell ablation cases (Fig 3J). In small islets (<89 μm diameter) while the islet-average [Ca^2+^] influx decreased following ablation of each cell type, the decrease was significantly greater following ablation of first responder cells. Consistent with these observations the proportion of cells that showed elevated first-phase [Ca^2+^] (i.e., within 5 min of [Ca^2+^] elevation) was largely unchanged in large islets, with 93% of cells showing elevated [Ca^2+^] for ablation of both first responder or control cells (Fig 3K). In small islets, the proportion of cells that showed elevated first-phase [Ca^2+^] was largely unchanged following control cell ablation (91% of cells), but substantially decreased following first responder cell ablation (42% of cells). A similar trend was observed in terms of connectivity during the first-phase [Ca^2+^] elevation: The mean number of functional links within the islet was significantly less following first responder cell ablation compared to the control cell ablation (Fig 3L). Finally, we measured the response time of the islet following ablation of a first responder cell (Fig 3M and 3N). We did not observe significant changes in large islets under ablation of a first responder cell, control cell, or with no ablation. Similarly, we did not observe significant change in small islets under ablation of a control cell or with no ablation. However, following ablation of a first responder cell, the time for a [Ca^2+^] response of an islet significantly increased (Fig 3M and 3N). Thus, in smaller islets, removal of a first responder cell via femtosecond laser ablation diminishes and delays the elevation of first-phase [Ca^2+^] as a result of there being fewer cells that respond both rapidly and in a coordinated fashion.

### First responder role depends on membrane excitability and islet context

**Fig 4:**
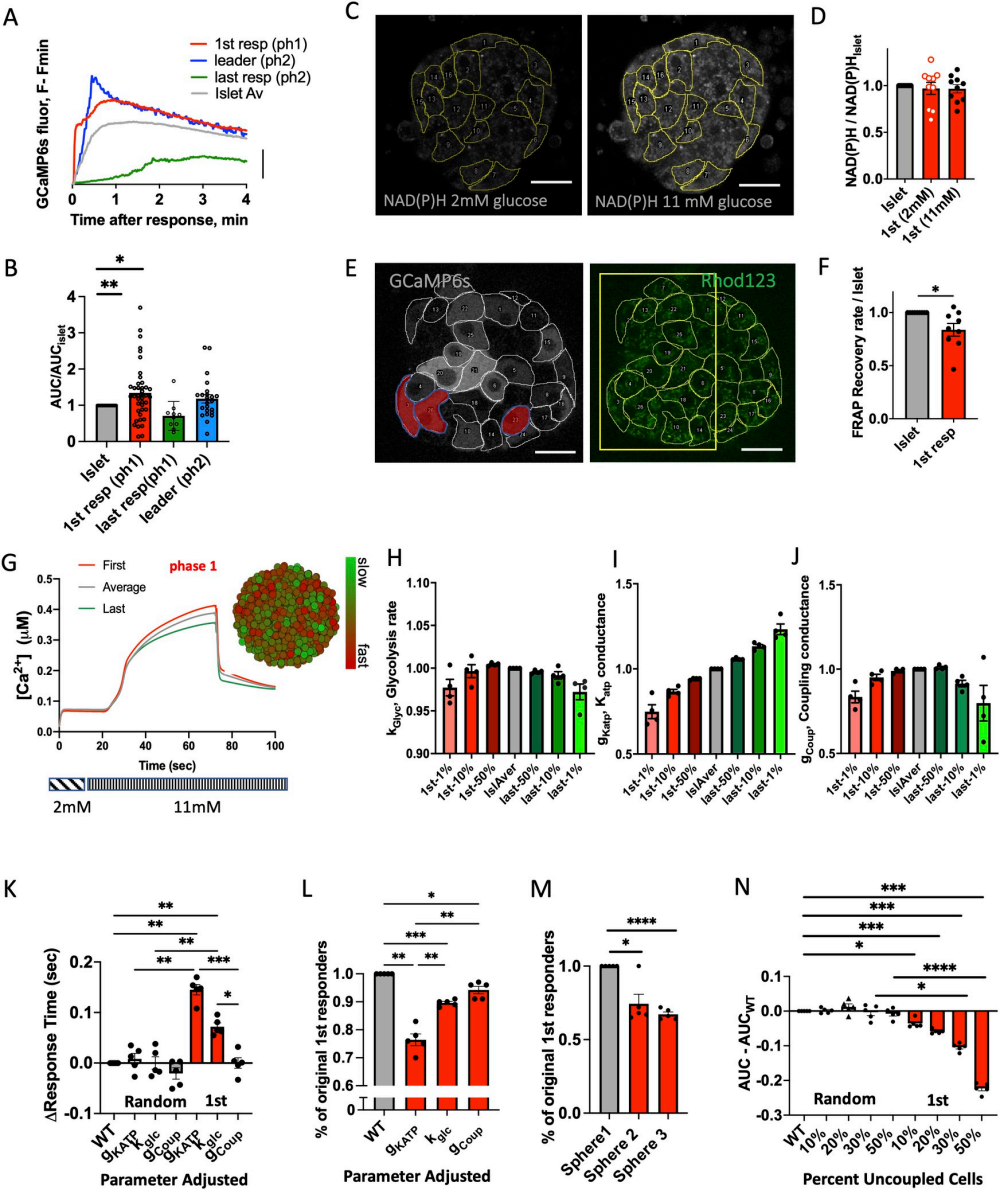
Characteristics of first responder cells. (**A**) Time course of [Ca^2+^] elevation in each cell state, indicating AUC. (**B**) Quantification of the AUCs shown in (A) for each cell state during the first 3 min after [Ca^2+^] elevation upon elevated glucose (16 islets from 9 mice). (**C**) Representative image of NAD(P)H autofluorescence at low (2 mM) and high (11 mM) glucose. (**D**) Mean NAD(P)H intensity in each identified subpopulation of β-cell, at low (2 mM) and high (11 mM) glucose, normalized with respect to the islet average (*n* = 10 islets from 10 mice). (**E**) Reprehensive GCamP6s fluorescence and Rhodamine123 fluorescence (for FRAP measurements) within the same islet z-plane (cell layer). (**F**) Mean Rhodamine123 fluorescence recovery rate as calculated during FRAP (*n* = 7 islets from 5 mice). Scale bar indicates 20 μm. **Modeling first responder cells.** (**G**) Representative time course (first phase only) of [Ca^2+^] in a simulated islet, indicating the [Ca^2+^] dynamics characterizing the first responder (1st-resp) and last responder (last-resp) cells compared to the islet average. (**G insert**) False-color map indicating [Ca^2+^] response time to glucose elevation during the first phase. (**H**) Mean parameter value of glycolysis rate (k_glyc_) averaged over the first (red) and last (green) indicated % of responding cells (*n* = 4 seeds). Data are normalized with respect to the islet-average parameter. (**I**) As in (H) for K_ATP_ conductance (g_KATP_). (**J**) As in (H) for coupling conductance (g_coup_). (**K**) Change in response time upon adjustment of the glycolysis rate (k_glyc_), K_ATP_ conductance (g_KATP_), and coupling conductance (g_coup_) in first responder, or in a random subset of cells. (**L**) Percent of the original first responder cells that remain among the earliest responders after adjustment of k_glyc_, g_KATP_, g_coup_ in first responder cells. (**M**) Percent of the original first responders that remain among the earliest responders after adjustment of the spatial position of the first responders in 3 separate distributions (spheres) (*n* = 5 seeds). (**N**) Decrease in the AUC of the first phase of the Ca^2+^ response following de-coupling (removal) of a given % of the first responder or a random set of cells from the islet (*n* = 5 seeds). Statistical analysis in C utilized 1-sample *t* test, where **** represents *p* < 0.0001, *** *p* < 0.0002, ** *p* < 0.0021, * *p* < 0.0332 for comparison of the islet-average value vs. all other groups. In D, F utilized 1-sample *t* test (with the null hypothesis of 1st responder difference from the islet being 0), where * *p* < 0.05 indicated comparing the islet-average value vs. first responder group. Statistical analysis in K–N utilized 1-way ANOVA (with Tukey’s multiple comparison post hoc test), where **** represents *p* < 0.0001, *** *p* < 0.001, ** *p* < 0.01, * *p* < 0.05 comparing the groups indicated. See S4 Data file for values used in each graph. AUC, area under the curve.

We next examined characteristics of the first responder cells that may allow them to show an earlier response time. β-cells that have been previously identified to disproportionately recruit or maintain elevated [Ca^2+^] in neighboring cells have shown elevated excitability, such as arising from increased metabolic activity [22]. As we showed earlier, the glibenclamide-induced membrane depolarization and subsequent calcium response was earlier in the cells that also had earlier responses to glucose. This suggests that first responder cells are more excitable. We then examined the [Ca^2+^] influx upon glucose stimulation (Fig 4A). First responder cells demonstrated significantly higher influx of [Ca^2+^] during the first-phase [Ca^2+^] response (*p* = 0.0072), while last responder cells had significantly lower [Ca^2+^] influx (*p* = 0.453) (Fig 4B). First responder cells did not have a greater than average NAD(P)H levels at either low (2 mM) or elevated (11 mM) glucose (Fig 4C and 4D), indicating glucose metabolism was not a distinguishing feature of first responder cells. Interestingly, following measurement of dye transfer kinetics via FRAP, first responder cells showed lower than average gap junction permeability (*p* = 0.0270) (Fig 4E and 4F).

To further understand which characteristics of first responder cells are required for their action, we utilized a previously published multicell model of islet β-cell electrophysiology [34]. This model incorporates heterogeneity in multiple metabolic, electrical, and gap junctional characteristics. We simulated the [Ca^2+^] response upon elevated glucose (Fig 4G) and observed significant variability in the time of [Ca^2+^] elevation (Fig 4G insert). We defined first responders as the 10% of all cells with the fastest response times, as in experiments. We first examined the characteristics of these first responder cells in the model islet. The rate of glycolysis for model first responder cells was not significantly different from the islet average (Fig 4H), consistent with experimental NAD(P)H measurements. The total open channel K_ATP_ conductance (equivalent to the number of channels in the plasma membrane) for model first responder cells was significantly lower than the islet average (Fig 4I), in agreement with the experimental earlier-than-average response time under glibenclamide stimulation. The coupling conductance for model first responder cells was slightly, but significantly lower than the islet average (Fig 4J), consistent with experimental gap junction permeability measured via FRAP. Model last responder cells showed no difference in glycolysis rate, elevated K_ATP_ conductance and reduced coupling conductance (Fig 4H–4J). Thus, model first responder cells are characterized by a combination of both lower electrical coupling and higher membrane excitability because of lower K_ATP_ conductance, whereas model last responder cells are characterized by a combination of both lower electrical coupling and lower membrane excitability because of greater K_ATP_ conductance.

To investigate the relative role of the abovementioned parameters (glycolysis rate, K_ATP_ conductance, electrical coupling) in the functional role of the first responder cells, we adjusted each of them to be equal to the corresponding islet-average parameter, basically forcing first responders to be no different from the islet-average cell (S6 Fig). If a specific parameter is necessary for the function of the first responder, adjusting that parameter would be expected to significantly change the response time of that cell. Of the 3 parameters examined, adjusting the K_ATP_ conductance had the greatest impact on the first responder cell response time (Fig 4K) and whether a first responder cell retained the earliest [Ca^2+^] elevation (Fig 4L). In addition, rearrangement of the cell position within the islet, and thus the neighboring cells in contact with the first responder cell, significantly disrupted whether a first responder cell showed the earliest elevation in [Ca^2+^] (Fig 4M). Model first responder cells are defined by both increased excitability and position within the islet (i.e., which neighboring cells are surrounding and coupled to the first responder cell).

Finally, we tested whether first responder cells acting via gap junction coupling were sufficient to recruit neighboring cells. Following simulation of the islet, we determined first responder cells. We then removed either a random set of cells (control) or the earliest responding cells. We then we re-simulated the islet [Ca^2+^] response to determine the effect of removal of these cells. Consistent with experimental measurements (ablation experiments), removal of first responder cells diminished the AUC for the islet-average [Ca^2+^] elevation, whereas removing a control set of cells had no impact on the islet-average [Ca^2+^] elevation (Fig 4N). Importantly, a decrease in [Ca^2+^] elevation was only observed for removal of greater than 10% of the earliest responding cells. The higher % of cells removed lead to a greater reduction of the [Ca^2+^] influx into the islet. Thus, since our model assumed that the only cell–cell interactions were gap junction coupling, we showed that gap junctional interaction is sufficient for a population of first responder cells to exert control over first-phase [Ca^2+^] in the islet.

## Discussion

β-cells within the islet are functionally heterogeneous. While subpopulations of β-cells have been suggested to maintain coordinated oscillatory [Ca^2+^] and insulin release [35], these are associated with the second phase of the [Ca^2+^] response; a point at which cell–cell electrical communication is less important for recruiting [Ca^2+^] and the level of insulin release, but is critically important for coordinating pulsatile [Ca^2+^] and insulin secretion dynamics. Given the importance of cell–cell electrical communication in regulating first-phase insulin release [16], we examined whether there exists a subpopulation of β-cells associated with the first-phase calcium response. We discovered a functional cell state that we termed “first responder.” First responder cells lead the first-phase [Ca^2+^] response and were distinct from previously identified functional subpopulations of β-cells. These cells were more excitable, critical for recruiting β-cells to elevate [Ca^2+^] immediately following glucose stimulation (S7 Fig). We further demonstrated that this state of the β-cell is conserved over an approximately 24 h time period.

### First responder cells represent a distinct functional state of the β-cell

We defined first responder cells as those β-cells that lead the first-phase [Ca^2+^] response, more specifically the 10% of cells in the islet with the earliest response time. Importantly upon repeated stimulation, the same cells generally showed an earlier-than-average response over 1 to 2 h (Fig 2), unlike last responder cells, which lacked any consistency. Only in approximately 50% of the islets initial first responder cells remained consistent during repeated glucose elevation (S5A Fig). Therefore, the temporal consistency of this cell state is not rigid. Some of them surrendered their first responder role either to a nearest neighbor cell or to a 2nd nearest neighbor. Rather, the spatial location of the first responder cluster remained consistent (Fig 2C).

We demonstrated that first responder cells are distinct from other functional subpopulations of β-cells previously defined by [Ca^2+^] signatures. Leader (wave origin) sells have been associated with regulating second-phase [Ca^2+^] dynamics [22,23]. Hub cells have been associated with maintaining elevated, coordinated [Ca^2+^]. Thus, the heterogeneity that controls second-phase [Ca^2+^] is different than the first-phase [Ca^2+^]. We do note that hub cells were previously identified following more rapid measurements of [Ca^2+^] dynamics (approximately 10 fps) than in our study here (approximately 1 fps) [20]. The lack of fast (<1 s) timescale [Ca^2+^] dynamics in our analysis may therefore exclude some hub cells and mean that the hub-like (ph2) cells we identify are not exactly analogous to those previously identified. Further, we studied the slow oscillations in this work, while previous studies looked at fast (<60 s period) [Ca^2+^] oscillations.

While the first responder cells remained in the lead over 1 to 2 h, this consistency was gradually lost after 24 h (Fig 2K). The overall responsiveness of the islet was maintained (demonstrated by calcium influx, Fig 2J), indicating the loss of state is not simply due to islet dysfunction. This finding suggests that first responder cells represent a transient functional state of the cell and not a permanent subpopulation of the β-cell. We are not aware of other long-term imaging studies beyond 1 to 2 h that test whether functional subpopulations represent transient states of the β-cell rather than permanent β-cell subpopulations. The current lack of genetic markers for first responder cells hinders robust lineage tracing approaches to validate this finding. Indeed, this first responder cell state may represent a subset of the more mature functional cell subpopulations that have been genetically marked [2]. Cyclic expression of *Ins2* gene activity has been reported in subsets of β-cells [36], suggesting that transient states of the β-cell can exist. A correlation between *Ins* expression and *GJD2* (coding Cx36) expression has also been demonstrated [32].

Thus, we speculate that fluctuations in Cx36 expression may contribute to first responder cells: increases in Cx36 gap junction conductance would suppress first responder cells as a result of hyperpolarization by less excitable neighboring cells. Decreases in Cx36 gap junction conductance (as is observed in first responder cells, Fig 4F) would allow the more excitable first responder cells to respond earlier and impact their neighboring cells. Decreased gap junction conductance would prevent a cell being hyperpolarized and inhibited by less excitable neighboring cells. Our computational model of the islet also showed lower gap junction conductance within the first responder cells. However, we cannot exclude a role for intra-islet paracrine factors in also regulating first responder cells.

An important consideration is that our study, along with other studies [20,22], examines a single plane of cells within the islet. Thus, the first responder cells are relative to that islet region. It is quite likely that elsewhere in the islet at other planes, cells that respond earlier are present which may play a more important role. Nevertheless, first responder cells represent a distinct functional state of the β-cell, stable for approximately 24 h and regulating the first phase of [Ca^2+^] and likely also insulin secretion. Whether in vivo this state is more or less stable in time still remains to be tested.

### First responder cells represent a more excitable subpopulation

We hypothesized and demonstrated that first responder cells are important to recruit elevated [Ca^2+^] in neighboring cells. Previously, we identified highly excitable cells that effectively recruited elevated [Ca^2+^] in neighboring cells [22]. Those cells showed increased NAD(P)H responses. Unlike previously reported highly excitable cells, first responder cells did not show significant difference in NAD(P)H levels (Fig 4D). First responder cells however did show increased [Ca^2+^] influx upon glucose stimulation compared to neighboring cells (Fig 4B) and some level of consistency following glibenclamide stimulation that inhibits K_ATP_ channels (Fig 2H), although not to the degree found with glucose stimulation. First responder cells likely show differences in ion channel composition, which may include reduced K_ATP_, but equally could include an increased resting depolarizing current. We illustrate this concept in S7A Fig. For example, increased expression of HCN channels has been found in a population of human beta-cells that is important for increased insulin secretion [37]. The reduced correlation between glucose and glibenclamide stimulation suggests that first responders are also defined by other factors in addition to higher electrical membrane excitability. Functional subpopulations (e.g., “hub cells,” “wave origin,” or ‘leader’) have been identified to show differences in glucose metabolism [20,22], which may also explain their distinction from first responder cells.

Notably, we observed consistent results within our computational model of the islet (Fig 4). In the model, first responder cells showed decreased K_ATP_ conductance but little difference in metabolic (glucokinase) activity. Adjusting K_ATP_ conductance in the first responder cells also led to original first responder cells losing their role. This further supports that first responder cells are more excitable due to altered ion channel composition. We also observed in the model that the cell position within the islet was important: rearranging the position of the first responder cells, and thus the neighboring cells in contact with the first responder cells, disrupted their time of response. Our finding that the first-phase response time is spatially organized further suggests the spatial positioning, and thus the influence of neighboring cells within this region is important (S7C Fig).

### First responder cells drive first-phase [Ca^2+^] elevation

Following femtosecond laser ablation of first responder cells, we observed a decline in the first-phase [Ca^2+^] response: a lower level of [Ca^2+^] uptake, decreased numbers of cells showing elevated [Ca^2+^], decreased [Ca^2+^] coordination and a longer response time from the glucose challenge (Fig 3), compared to the laser ablation of the non-first responder (control) cell. This indicates that first responder cells are necessary for both recruiting and coordinating elevated [Ca^2+^] following glucose elevation. Without first responder cells, the islet is slower to respond to glucose, and the response is weaker and less organized. We observed qualitatively similar results in the islet model after removal of first responder cells: The integrated [Ca^2+^] elevation over the first phase was diminished (Fig 4N). Since model only includes gap junction interaction between the beta-cells, the match between theory and experiment indicates that gap junction coupling is sufficient to mediate the impact of first responder cells over the islet first-phase [Ca^2+^] response to glucose. At least in the mouse islets, where beta-cells mostly interact with each-other and not with alpha, delta, and other cells.

In zebrafish islets, laser ablation of those cells that lead the [Ca^2+^] elevation in response to glucose also disproportionately affected islet-average [Ca^2+^] influx, as compared to laser ablation of control cells [24]. Zebrafish islets in that study were comparable in size to the small mouse islets analyzed in our work. It is possible that these zebrafish “leader” cells are analogous to the first responder cells in mouse islets that we describe in this study.

Given that different cells are leading different phases of the [Ca^2+^] response to glucose (first responders– 1st phase versus leader cells– 2nd phase), we conclude that first responder cells are more important for recruiting and coordinating [Ca^2+^] during a specific time window, when the glucose is changed from low to high (S7D Fig). Interestingly, this observation is similar to the regulation of [Ca^2+^] by Cx36 gap junction channels. At elevated glucose, almost all β-cells are capable of elevating [Ca^2+^] and insulin release. However, some cells elevate [Ca^2+^] and insulin release more rapidly and some elevate more slowly, likely as a result of being more or less excitable, respectively. As a result, while all cells elevate [Ca^2+^], the differences in their timing prevent a robust first-phase insulin release [16].

While model results presented here indicated that gap junction coupling was sufficient for 10% of first responder cells to drive first-phase [Ca^2+^], we do note that more substantial diminishments of [Ca^2+^] influx were observed when greater numbers of earlier responding cells were removed. This suggests the existence of functional redundancy in the ability of earlier responding cells to drive the first-phase [Ca^2+^] elevation. Indeed, following ablation of a first responding cell, the “new” first responding cells were cells that responded earlier than average prior to cell ablation. This further explains how larger islets were relatively resistant to destruction of individual cells. Measuring how loss of first responder cells impacts insulin release itself will also be important to establish, particularly to establish whether changes in first-phase [Ca^2+^] impact first-phase insulin release.

## Summary

Several functional β-cell subpopulations have been identified that influence islet function, yet it is currently unknown whether these subpopulations overlap and whether the mechanisms by which they affect the islet function are the same. This understanding has been hindered by a lack of standard procedures in identifying functional subpopulations (e.g., by standard analysis of [Ca^2+^] dynamics, optogenetic stimulation, or silencing). Longer-term imaging to track the consistency and state of functional subpopulations has also been missing. We combined high-resolution confocal microscopy in islets with β-cell-specific [Ca^2+^] sensor expression, together with targeted removal of single cells via 2-photon laser ablation and standardized analysis of [Ca^2+^] coordination. We discovered a distinct functional β-cell state that was stable for approximately 24 h. The state is characterized by increased electrical excitability and slightly reduced gap junction permeability. This state did not overlap with other previously identified functional subpopulations. We discovered organization of the first-phase [Ca^2+^] response and existence of hierarchy in this response where the second earliest to response cell takes over the role of the first responder cell upon the first responder cell ablation. Removal of first responder cells disproportionately disrupted the response time of the islet and [Ca^2+^] levels during the first phase following glucose elevation, compared to the control cell removal in the size-matched islets. Thus, we have identified a cell state that is functionally important to a first phase of calcium response to glucose in individual islets.

## Materials and methods

### Animal care

Male and female mice were used under protocols approved by the University of Colorado Institutional Animal Care and Use Committee (IACUC Protocol number: 00024). β-cell-specific GCaMP6s expression (β-GCaMP6s) was achieved through crossing a MIP-CreER (The Jackson Laboratory) and a GCaMP6s line (The Jackson Laboratory). Genotype was verified through qPCR (Transetyx, Memphis, Tennessee, United States of America). Mice were held in a temperature-controlled environment with a 12 h light/dark cycle and given continuous access to food and water. CreER-mediated recombination was induced by 5 daily doses of tamoxifen (50 mg/kg bw in corn oil) delivered IP.

### Islet isolation and culture

Islets were isolated from mice under ketamine/xylazine anesthesia (80 and 16 mg/kg) by collagenase delivery into the pancreas via injection into the bile duct. The collagenase-inflated pancreas was surgically removed and digested. Islets were handpicked and planted into the glass-bottom dishes (MatTek) using CellTak cell tissue adhesive (Sigma-Aldrich). Islets were cultured in RPMI medium (Corning, Tewksbury, Massachusetts, USA) containing 10% fetal bovine serum, 100 U/mL penicillin, and 100 mg/mL streptomycin. Islets were incubated at 37°C, 5% CO2 for 24 to 72 h before imaging.

### Imaging

An hour prior to imaging nutrition media from the isolated islets was replaced by an imaging solution (125 mM NaCl, 5.7 mM KCl, 2.5 mM CaCl2, 1.2 mM MgCl2, 10 mM HEPES, and 0.1% BSA (pH 7.4)) containing 2 mM glucose. During imaging, the glucose level was raised to 11 mM. Islets were imaged using either an LSM780 system (Carl Zeiss, Oberkochen, Germany) with a 40× 1.2 NA objective or with an LSM800 system (Carl Zeiss) with 20× 0.8 NA PlanApochromat objective or a 40× 1.2 NA objective, with samples held at 37°C.

For [Ca^2+^] measurements, GCaMP6s fluorescence was excited using a 488-nm laser. Images were acquired at 1 frame/s at 10 to 15 μm depth from the bottom of the islet. Glucose was elevated 3 min after the start of recording, unless stated otherwise.

NAD(P)H autofluorescence and [Ca^2+^] dynamics were performed in the same z-position within the islet. NAD(P)H autofluorescence was imaged under 2-photon excitation using a tunable mode-locked Ti:sapphire laser (Chameleon; Coherent, Santa Clara, California, USA) set to 710 nm. Fluorescence emission was detected at 400 to 450 nm using the internal detector. Z-stacks of 6 to 7 images were acquired spanning a depth of 5 μm. First, the NAD(P)H was recorded at 2 mM glucose, then the [Ca^2+^] dynamics was recorder at 2 mM and during transition to 11 mM glucose. After the [Ca^2+^] wave was established, the NAD(P)H was recorded at 11 mM glucose.

Cx36 gap junction permeability and [Ca^2+^] dynamics were performed in the same z-position within the islet, with gap junction permeability measured using fluorescence recovery after photobleaching, as previously described [17]. After [Ca^2+^] imaging, islets were loaded with 12 mM Rhodamine-123 for 30 min at 37°C in imaging solution. Islets were then washed and FRAP performed at 11 mM glucose at room temperature. Rhodamine-123 was excited using a 488-nm laser line, and fluorescence emission was detected at 500 to 580 nm. Three baseline images were initially recorded. A region of interest (ROI) was then photobleached achieving, on average, a 50% decrease in fluorescence, and images were then acquired every 5 to 15 s for 15 min.

### Imaging long-term [Ca^2+^] dynamics

The initial [Ca^2+^] dynamics under glucose elevation from 2 to 11 mM at 0 h was recorded. The dish was marked to indicate its orientation with respect to the microscope stage, and the arrangement of islets was noted to facilitate islet localization in subsequent imaging. After this first time point, imaging solution was replaced by the islet culture media, and the dish was kept in the incubator at 37°C and 5% CO2 until the next time point. The same cell layer in the islet was imaged at 6 h intervals until 48 h. For some islets intervals of 12 h were recorded.

### Laser ablation

Laser ablation was performed with 2-photon tunable mode-locked Ti:sapphire laser (Chameleon; Coherent, Santa Clara, California, USA) set to 750 nm. The power at objective was 150 mW. First [Ca^2+^] dynamics were recorded at 2 mM and 11 mM glucose, and first responder cells were identified. Then, glucose was lowered to 2 mM and [Ca^2+^] activity was monitored to ensure the islet returns to a basal level of activity. The first responder cell(s) were identified, and a sub-cell-sized ROI to be ablated (5 × 5 μm) was selected either over the first responder cell or over a control cell far from the first responder. Ablation was performed by illuminating the ROI. [Ca^2+^] dynamics were then imaged during the transition from 2 mM to 11 mM glucose.

### Analysis of [Ca^2+^] dynamics

We defined first responder cells as 10% of the cells imaged within an islet that responded earliest to show elevated [Ca^2+^]. The time of response was defined as time at which the intensity of the fluorescence of the [Ca^2+^] indicator (GCaMP6s) reached half the maximum height following islet stimulation by glucose or other secretagogue. We refer to this time as half-height time or response time throughout the text.

The leader and lagger cells were defined during the second-phase [Ca^2+^] response to glucose, once oscillations emerge. These cells were defined based on the phase lag of the slow [Ca^2+^] oscillation in each cell with respect to the phase of the average [Ca^2+^] oscillation across the islet, as previously presented [38]. Islets that lacked second-phase [Ca^2+^] oscillations were excluded from the analysis of the oscillatory phase and only used for first-phase analysis.

For analysis of long-term [Ca^2+^] dynamics, only those cells that correspond to cells imaged in the first time point (0 h) were considered. These cells were identified by locating their relative positions with respect to non-β-cells that did not express GCaMP6s. Additional cells that appear in the plane of as a result of islets slowly flattening over time were not considered.

### Distances between the cells

X and Y coordinates of centers of each ROI outlining a cell were obtained in Fiji software. Then, distances between the cells of interest were calculated using these coordinates. In Fig 1J distance from the first responder to the first responder indicates the average distance between all first responder cells per islet. For example, in the islet shown in Fig 1I, there were several first responding cells (cell #30, #34, …, #48), the distances were measured from cell #48 to #30, from #30 to #34, etc. And then, the average value was plotted as a dot in Fig 1E (red bar). Similarly, distances between each first responder and other cells of interest were calculated and averaged per islet.

### Network analysis

Network connectivity analysis for second-phase [Ca^2+^] response presented in Figs 1 and S1 was performed by Dr. David Hodson as he reported previously [20] and using the same algorithm. Briefly, [Ca^2+^] time courses were binarized based on intensity deviation from the mean intensity using 20% intensity cutoff. Intensity above this cutoff was assigned a value of 1, and below—value of 0. A co-activity matrix with elements C_ij_ for each cell pair in the islet was constructed from the binarized signal.


$$C_{ij}={\frac{T_{ij}}{\sqrt{T_{i}T_{j}}}}_{}$$ (1)

The T_i_ and T_j_ represent time (sec) of activity (when intensity was >20% cutoff) for cells *i* and *j*, and the T_ij_ represents time of co-activity of a cell pair. Then, the co-activity matrix was shuffled >9,999 times to construct a random co-activity matrix, with elements C_ij_*, which was used to account for a co-activity being due to chance. The experimental co-activity matrix was then adjusted using threshold constructed of the mean value of C_ij_* and a standard deviation from the mean, σ*:

$${Thr}_{ij}={{C_{ij}}^{*}+{2\sigma }^{*}.}_{}$$ (2)

The percent of links was calculated with respect to the maximum number of links per cell in each individual islet. For example, if a most connected cell possessed max = 10 links and other cells had 1, 3, …7—then % were: 10%, 30%, …70%.

Pearson-product-based network analysis presented in Fig 3L was performed as previously reported [21]. [Ca^2+^] time courses were analyzed during the first-phase [Ca^2+^] response, and the analyzed time ranges were chosen to be equal for pre- and post-ablation. The Pearson product for each cell pair in the islet was calculated over each time point, and the time-average values were computed to construct a correlation matrix. An adjacency matrix was calculated by applying a threshold to the correlation matrix. The same threshold of 0.9 was applied to all islets. All cell pairs with non-zero values in the adjacency matrix were considered to have a functional link.

### Islet modeling

The coupled β-cell model was described previously [34] and adapted from the published Cha–Noma single-cell model [39,40]. All code was written in C++ and run on the SUMMIT supercomputer (University of Colorado Boulder).

The membrane potential (V_i_) for each β-cell i is related to the sum of individual ion currents as described by [39]:

$$C_{m}\frac{dV_{i}}{dt}=I_{Cav}+I_{TRPM}+I_{SOC}+I_{bNSC}+I_{KDr}+I_{KCa(SK)}+I_{K_{ATP}}+I_{NaK}+I_{NaCa}+I_{PMCA}+I_{NaCa}+I_{Coup}.$$ (3)

Where the gap junction mediated current I_Coup_ [19] is:

$$I_{Coup}=\sum_{i}g_{Coup}^{ij}(V_{i}-V_{j}).$$ (4)

There are *N* = 1,000 cells in each simulation. Heterogeneity was introduced by randomizing multiple variables according to a Gaussian distribution (S1 Table). Heterogeneity in Cx36 gap junctions was modeled as a γ-distribution with parameters k = θ = 4 as described previously [22] and scaled to an average g_Coup_ between cells = 120 pS.

The flux of glycolysis J_glc_, which is limited by the rate of k_glc_ activity in the β-cell, is described as:

$$J_{glc}=k_{glc}∙f_{glc}∙([{Re}_{tot}]-[Re]).$$ (5)

Where k_glc_ is the maximum rate of glycolysis (equivalent to GK activity), which was simulated as a continuous Gaussian distribution with a mean of 0.000126 ms^−1^ and standard deviation of 25% of the mean. [Re_tot_] = 10 mM, the total amount of pyrimidine nucleotides. The ATP and glucose dependence of glycolysis (GK activity) is:

$$f_{glc}=\frac{1}{1+\frac{K_{mATP}}{{\left[ATP\right]}_{i}}}∙\frac{1}{1+{\left(\frac{K_{G}}{\left[G\right]}\right)}^{hgl}}.$$ (6)

Where [G] is the extracellular concentration of glucose, hgl is the hill coefficient, K_G_ is the half maximal concentration of glucose, and K_mATP_ is the half maximal concentration of ATP.

In simulations where a specific heterogenous parameter is adjusted, the first 100 responding cells were identified (see below), and these first responder cells were adjusted so that the parameter of choice (g_KATP_, k_glc_, or g_Coup_) was forced to be at the islet-average value (S1 Table). We then compensated a slight shift of the islet-average (g_KATP_, k_glc_, or g_Coup_) values, caused by this manipulation, by slightly adjusting them to match the original islet average. A new simulation with these adjusted parameters was run. As a control, a random set of 100 cells are distributed across the islet, and these random cells were selected with the same spatial organization as first responder cells from a simulation that initiated with a different random number of seed. These random cells are adjusted in the same way as described above to the islet-average parameter value.

### Islet modeling analysis

All simulation data analysis was performed using custom MATLAB scripts. The scripts are available from Github: https://github.com/jaelennox/FirstResponderCells_Kravetsetal along with the example files for the scripts.

First responder cells were determined using the [Ca^2+^] time courses of each cell during the first 20 to 72 s, which covers the first-phase [Ca^2+^] response. The response time was calculated as the time to half the maximum [Ca^2+^] level. First responder cells were then determined to be the 100 (10%) cells with the earliest response time.

In simulations where cells are uncoupled, a given % of cells with the lowest response time are uncoupled from the simulation, but the next 100 cells are analyzed as the first responders of this new simulation. To uncouple cells from the simulated islet, the conductance, g_Coup_, of the cells to be removed is set to 0 pS. Removed cells are excluded from subsequent islet analysis. For control simulations, a random % of cells are uncoupled from the islet. These cells are distributed across the islet in the same organization as the first responder cells from a different simulation.

### Statistical analysis

All statistical analysis was performed in Prism (GraphPad).

For computational results, a 1-way repeated measures ANOVA with Tukey post hoc analysis was utilized to test for significant differences between either the WT simulation or a “Random” control simulation that matched islets before and after parameters are adjusted or cells are uncoupled.

For experimental results, either 1-way ANOVA or a 1-sample *t* test were used (indicated in figure captions) to compare parameters of a specific β-cell subpopulation to the corresponding islet-average parameters. Data are reported as mean ± SEM (standard error in the mean) unless otherwise indicated. Linear regression analysis was presented as the trend with 95% confidence intervals. The difference of the slope from zero was evaluated with the F test and the *p* value was reported. For Figs 1F, 1G, 2K, and 2L, linear mixed-effects model (LMEM) was used with islet and mouse as random effects. The differences were considered significant when *p*-value < 0.05. The *Y*_*mic*_ in LMEM denotes the average distance from a cell to all 1st responders in the islet, and *X*_*mic*_ denotes the time of response for the *c*^*th*^ cell in the *i*^*th*^ islet in the *m*^*th*^ mouse.

- Model in Figs 1F, 1G, 2K, and 2L and all the models for Tresp, lags, links:
$$Y_{mic}=\beta_{0}+\beta_{1}X_{mi}+b_{mi}+e_{mic}.$$
- Model in S2 Fig:
$$Y_{mic}=\beta_{0}+\beta_{1}X_{mi}+b_{0mi}+b_{1mi}X_{mi}+e_{mic}.$$
  Where

- *β*_0_ is the fixed-effect intercept.
- *β*_1_ is the fixed-effect slope.
- *b*_0*mi*_ is the random-effect intercept.
- *b*_1*mi*_ is the random-effect slope.
- *e*_*mic*_ is the residual error.

All results of the LMEM are presented in the S2 Statistical Analysis LMEM file. The analysis was performed using R programing language.

### Sample size estimate

Power analysis was performed to estimate sample size required for each set of experiment. For example, for experimental set of the relative location of subpopulations (Fig 1D) mean and standard deviation of the distribution of distances from first responder cell to every other first responder in the same islet was chosen as the “expected” group. Power analysis yielded the *n* = 6 per group required to provide the effect size of 20% assuming symmetrical distribution. For the % of functional links (Fig 1), mean and standard deviation of the islet-median % of links distribution was chosen as the “expected” group. Power analysis yielded the *n* = 8 per group required to provide the effect size of 20% assuming symmetrical distribution. In the same fashion, mean and standard deviation of the pilot “expected” group were used for power analysis of the rest of the experimental sets (short- and long-term consistency of the response time of the 1st responder, difference in the electrical coupling measured with FRAP, effect of the laser ablation, etc.). The probability of the type 1 error, alpha was chosen as 5%, power was chosen as 80%, and the effect size of 20% was used to estimate sample size through the power analysis.

## Supporting information

S1 Fig (A) Cumulative response time (Tresp) distribution for 19 islets. (B) Location of 1st responder cells in the 2D islet plane, normalized by islet radius. (C) Representative false-color map of calcium phase lag. (D) Example of calcium leader and “lagger” cell time traces. (E and F) Link maps obtained using network analysis for first- and second-phase calcium response, correspondingly. (G and H) Adjacency matrices for first- and second-phase calcium response, correspondingly. (I) [Ca^2+^] response time to glucose elevation for different beta-cell states (*n* = 8–10 islets, m = 30–45 cells). (J) Phase lag of the Ca^2+^ wave with respect to the islet-average wave for different beta-cell states (*n* = 8–12 islets, m = 18–34 cells). (K and L) Coordination (network) analysis for *n* = 8 islets performed for first- and second-phase calcium dynamics, correspondingly. Functional network analysis was performed via binarization and co-activity matrix analysis, as described before [20]. Statistical tests: I, J: 1-sample *t* test, K, L: ordinary 1-way ANOVA, where **** represents *p* < 0.0001, *** *p* < 0.0002, ** *p* < 0.0021, * *p* < 0.0332 indicated for comparison of the groups. See **S5 Data** file for values used in each graph.
(PDF)

S2 Fig (A) Correlation between the absolute response time of each beta-cell in an islet and their proximity to the first responder beta-cell for 8 islets. See the Supporting information **S1 Example calculation** file. Solid line indicates regression, dashed line indicates 95% CI. (B) Examples of islets in which there are 2 domains with local first responder clusters per each domain, as well as distance of each cell in the islet plane to 1st responders (located in Domain1) vs. time of response to glucose. See S6 Data file for values used in each graph.
(PDF)

S3 Fig (A) Pseudo-color map representing time of response to glucose during the first-phase [Ca^2+^] response in 3 adjacent planes in the islet separated by 10 μm. First and last responder cells are highlighted with the white borders. (B) Pseudo-color map representing wave propagation in the same islet planes during the second-phase [Ca^2+^] response. Wave origin and wave end cells are highlighted with the white borders. (C and D) Plane-average positions of each beta-cell subpopulation for 3 planes. (E) Distances between the plane-average positions of each subpopulation (position in plane (i)–position in plane (j)). Average distances are approximately 20 μm, indicating that location of all 4 subpopulations of interest is conserved in 3D. (F) Polar angle between the plane-average positions of the subpopulations (angle in plane (i)–angle in plane (j)). Average angles are below 90 degrees, indicating spatial conservation of the location of all 4 subpopulations of interest in 3D. For (E and F) *n* = 4 islets were studied. See S7 Data file for values used in each graph.
(PDF)

S4 Fig (A) Quantification of the Ca^2+^ oscillation period and (B) frequency during initial and repeated glucose elevation (*n* = 4 islets). No significant difference was found in 1 sample *t* test with initial-repeated period (frequency) difference compared to 0. (C) Response time of all cells in the optical section of the islet during initial vs. during repeated glucose elevation. Red dots represent first responder and green represent last responder cells identified during the initial glucose elevation. See S8 Data file for values used in each graph.
(PDF)

S5 Fig (A) Left pie chart: Percent of islets in which initial first and last responders remain in their role during repeated glucose elevation. Middle pie chart: same as left but following control cell ablation. Right pie charts: same as middle but following first responder cell ablation. (B) Islet size dependence of the [Ca^2+^] influx into the islet (area under the curve of the [Ca^2+^] time course) for random and first responder ablation cases. (C) Size distribution of the islets studied in the ablation experiments. See S9 Data file for values used in each graph.
(PDF)

S6 Fig First responder parameter adjustment to be equal to that of the islet average. **(A)** Distribution of the glycolysis rate (k_glyc_) in non-first responders (gray) and first responders (red) before (left) and after (right) parameter adjustment. Adjustment was done to set first responder parameter to be the same as the islet-average value (*n* = 5 seeds). **(B)** As in (A) for adjustment of K_ATP_ conductance (g_KATP_). **(C)** As in (A) for adjustment of coupling conductance (g_coup_). See S10 Data file for values used in each graph.
(PDF)

S7 Fig Schematic representation of the role of first responder cells in the islet. (A) Cell1—first responder cell (red) is more prone to membrane depolarization upon glucose stimulation. Cell 2 (yellow) is less depolarized than a first responder. The difference in the membrane potential between the cell 1 and 2 leads to depolarizing current to flow through the gap junctions into cell 2 triggering depolarization. Cell 2 subsequently depolarizes the less excitable cell 3 (green). (B) Representation of the [Ca^2+^] response to glucose in cells 1,2,3 shown in (A) and the islet-average [Ca^2+^] response with (black) and without (gray) the first responder cell. (C) Schematic of the time of response in the islet (red—faster response, green—slower) before and after the first responder ablation. Post-ablation, the cell with the second earliest response time takes over the role of the first responder. (D) Representation of the [Ca^2+^] coordination before and dis-coordination after the first responder cell ablation.
(PDF)

S1 Table Values of heterogeneous parameters used in the islet simulations. (PDF)

S1 Data Values used in each graph of Fig 1. (XLSX)

S2 Data Values used in each graph of Fig 2. (XLSX)

S3 Data Values used in each graph of Fig 3. (XLSX)

S4 Data Values used in each graph of Fig 4. (XLSX)

S5 Data Values used in each graph of S1 Fig. (XLSX)

S6 Data Values used in each graph of S2 Fig. (XLSX)

S7 Data Values used in each graph of S3 Fig. (XLSX)

S8 Data Values used in each graph of S4 Fig. (XLSX)

S9 Data Values used in each graph of S5 Fig. (XLSX)

S10 Data Values used in each graph of S6 Fig. (XLSX)

S1 Statistical analysis LMEM LMEM (Linear mixed-effect model) analysis of the data presented in Figs 1F, 1G, and S1I and S1J. (DOCX)

S2 Statistical analysis LMEM Vira Kravets—mixed model—updates for PLOS BIO. (DOCX)

S3 Statistical analysis LMEM LMEM analysis of the data presented in S2 Fig. (DOCX)

S1 Example calculation Example calculations of the average distance to 1st responder vs. time of response to glucose. (XLSX)

S1 Code Import ROIs MATLAB Code to import cell ROIs for Ca analysis. Network analysis Fig 3L. MATLAB code for analysis of the Ca coordination (Fig 3L only). Ca Analysis MATAB Code. MATLAB routines used to analyze experimental Ca dynamics
(ZIP)

## Abbreviations

AUC: area under the curve
LMEM: linear mixed-effects model
ROI: region of interest
scRNAseq: single-cell RNA sequencing
