# Supplementary figures and images for: Functional architecture of pancreatic islets identifies a population of first responder cells that drive the first-phase calcium response
Source: PLoS Biol. 2022 Sep 13;20(9):e3001761. doi: 10.1371/journal.pbio.3001761 (PMC9506623; doi:10.1371/journal.pbio.3001761)

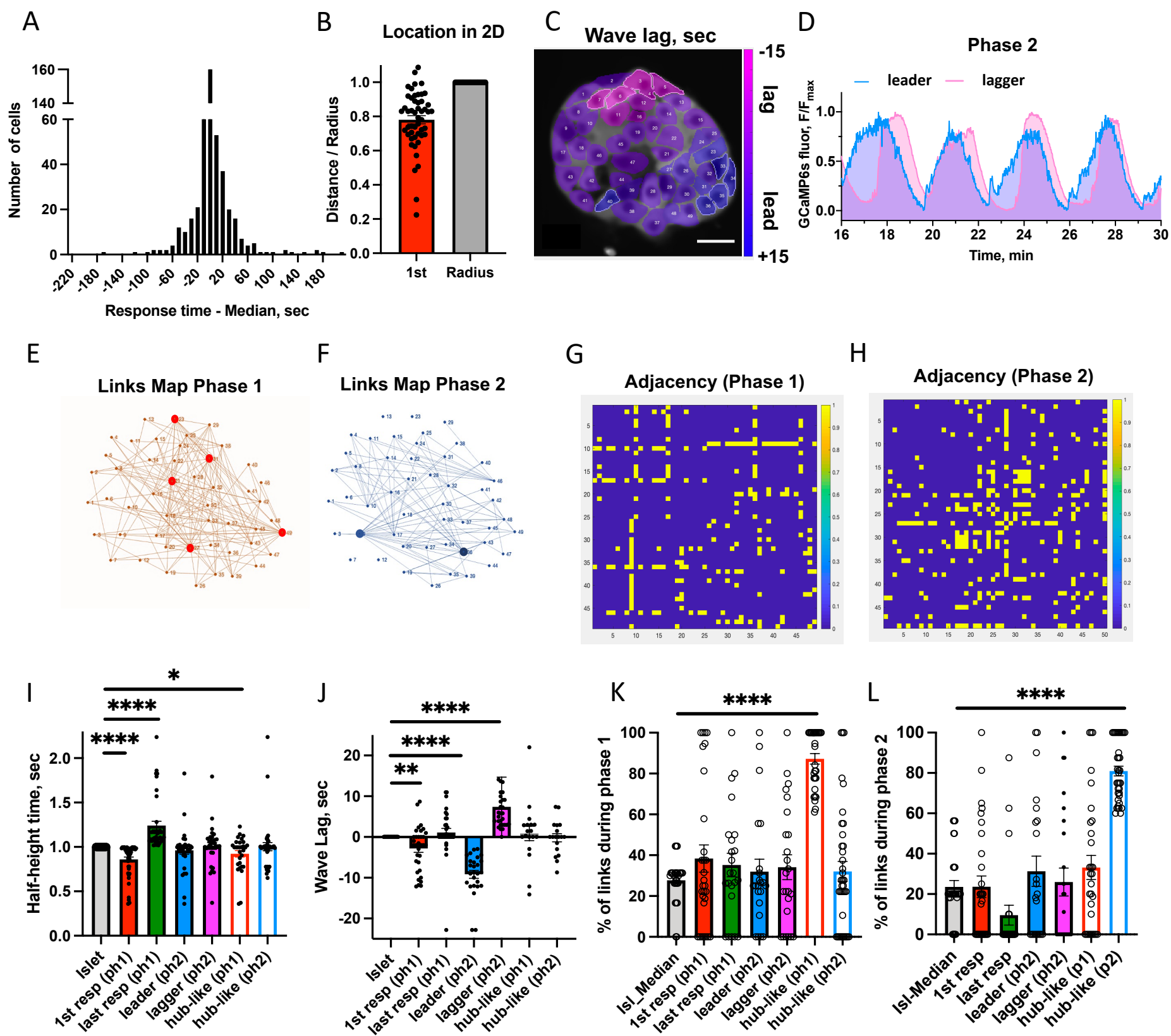

Figure S1

Supplement: S1 Fig — (A) Cumulative response time (Tresp) distribution for 19 islets. (B) Location of 1st responder cells in the 2D islet plane, normalized by islet radius. (C) Representative false-color map of calcium phase lag. (D) Example of calcium leader and “lagger” cell time traces. (E and F) Link maps obtained using network analysis for first- and second-phase calcium response, correspondingly. (G and H) Adjacency matrices for first- and second-phase calcium response, correspondingly. (I) [Ca2+] response time to glucose elevation for different beta-cell states (n = 8–10 islets, m = 30–45 cells). (J) Phase lag of the Ca2+ wave with respect to the islet-average wave for different beta-cell states (n = 8–12 islets, m = 18–34 cells). (K and L) Coordination (network) analysis for n = 8 islets performed for first- and second-phase calcium dynamics, correspondingly. Functional network analysis was performed via binarization and co-activity matrix analysis, as described before [20]. Statistical tests: I, J: 1-sample t test, K, L: ordinary 1-way ANOVA, where **** represents p < 0.0001, *** p < 0.0002, ** p < 0.0021, * p < 0.0332 indicated for comparison of the groups. See S5 Data file for values used in each graph. (PDF) [file pbio.3001761.s001.pdf]

A

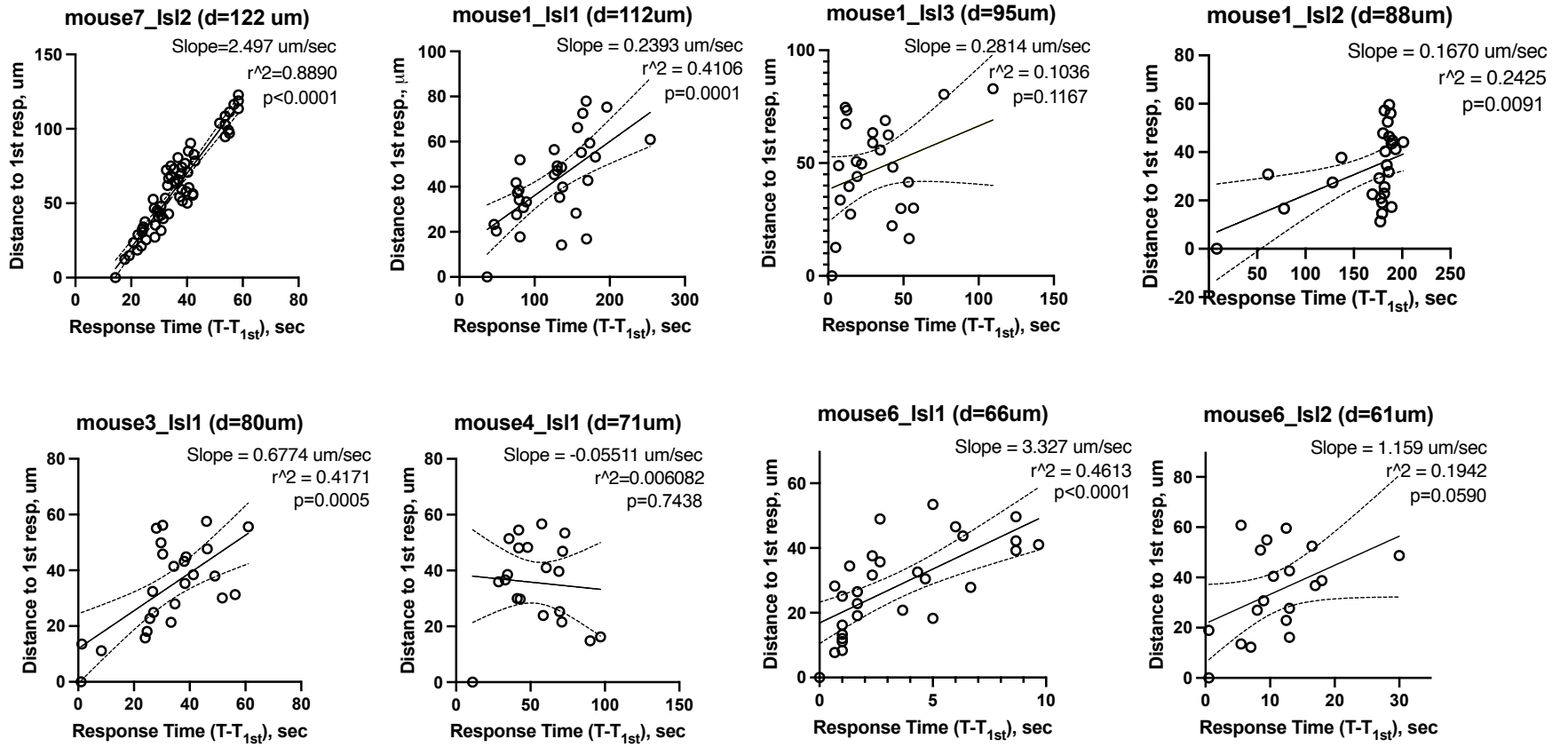

B

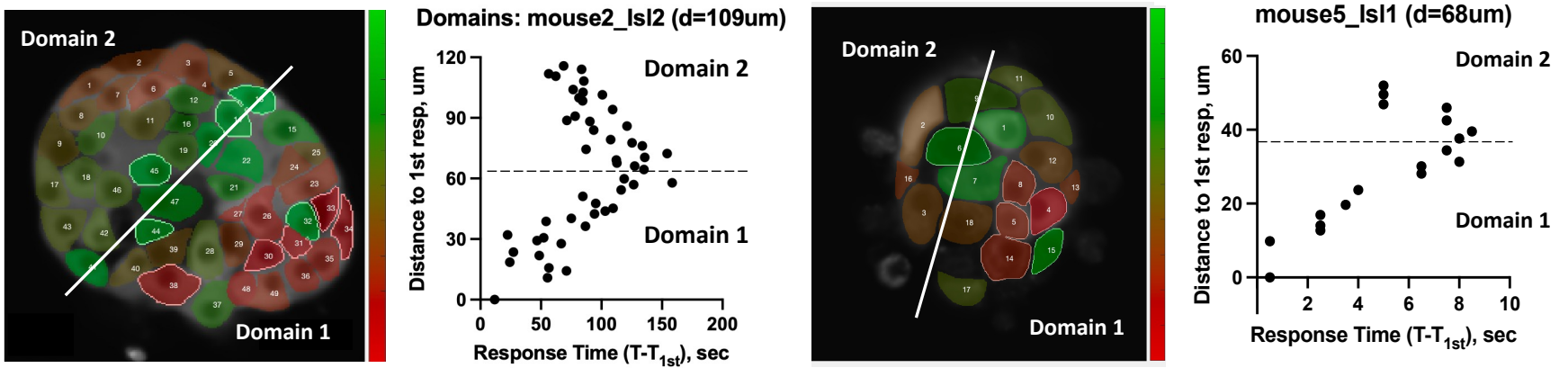

Figure S2

Supplement: S2 Fig — (A) Correlation between the absolute response time of each beta-cell in an islet and their proximity to the first responder beta-cell for 8 islets. See the Supporting information S1 Example calculation file. Solid line indicates regression, dashed line indicates 95% CI. (B) Examples of islets in which there are 2 domains with local first responder clusters per each domain, as well as distance of each cell in the islet plane to 1st responders (located in Domain1) vs. time of response to glucose. See S6 Data file for values used in each graph. (PDF) [file pbio.3001761.s002.pdf]

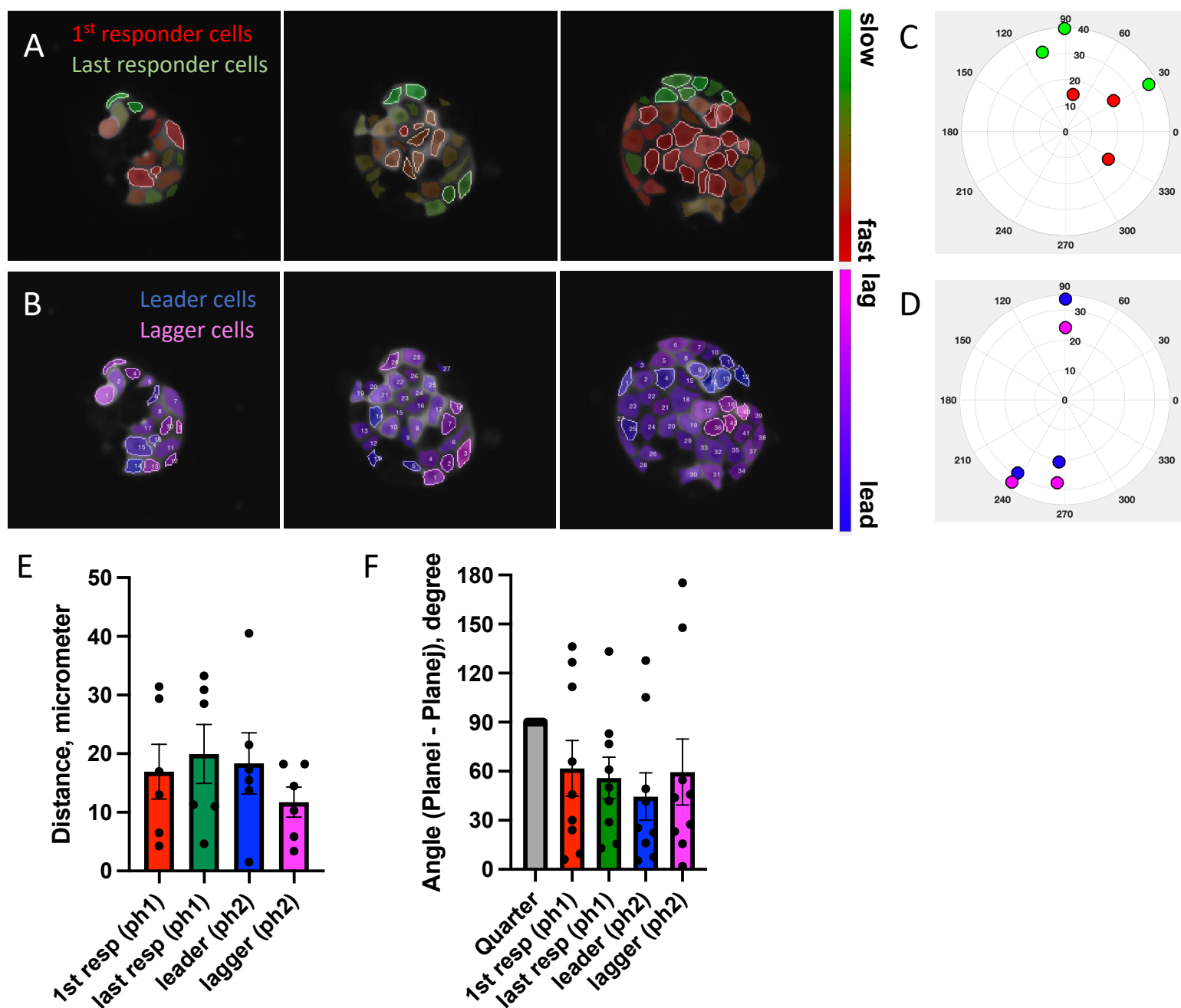

Figure S3

Supplement: S3 Fig — (A) Pseudo-color map representing time of response to glucose during the first-phase [Ca2+] response in 3 adjacent planes in the islet separated by 10 μm. First and last responder cells are highlighted with the white borders. (B) Pseudo-color map representing wave propagation in the same islet planes during the second-phase [Ca2+] response. Wave origin and wave end cells are highlighted with the white borders. (C and D) Plane-average positions of each beta-cell subpopulation for 3 planes. (E) Distances between the plane-average positions of each subpopulation (position in plane (i)–position in plane (j)). Average distances are approximately 20 μm, indicating that location of all 4 subpopulations of interest is conserved in 3D. (F) Polar angle between the plane-average positions of the subpopulations (angle in plane (i)–angle in plane (j)). Average angles are below 90 degrees, indicating spatial conservation of the location of all 4 subpopulations of interest in 3D. For (E and F) n = 4 islets were studied. See S7 Data file for values used in each graph. (PDF) [file pbio.3001761.s003.pdf]

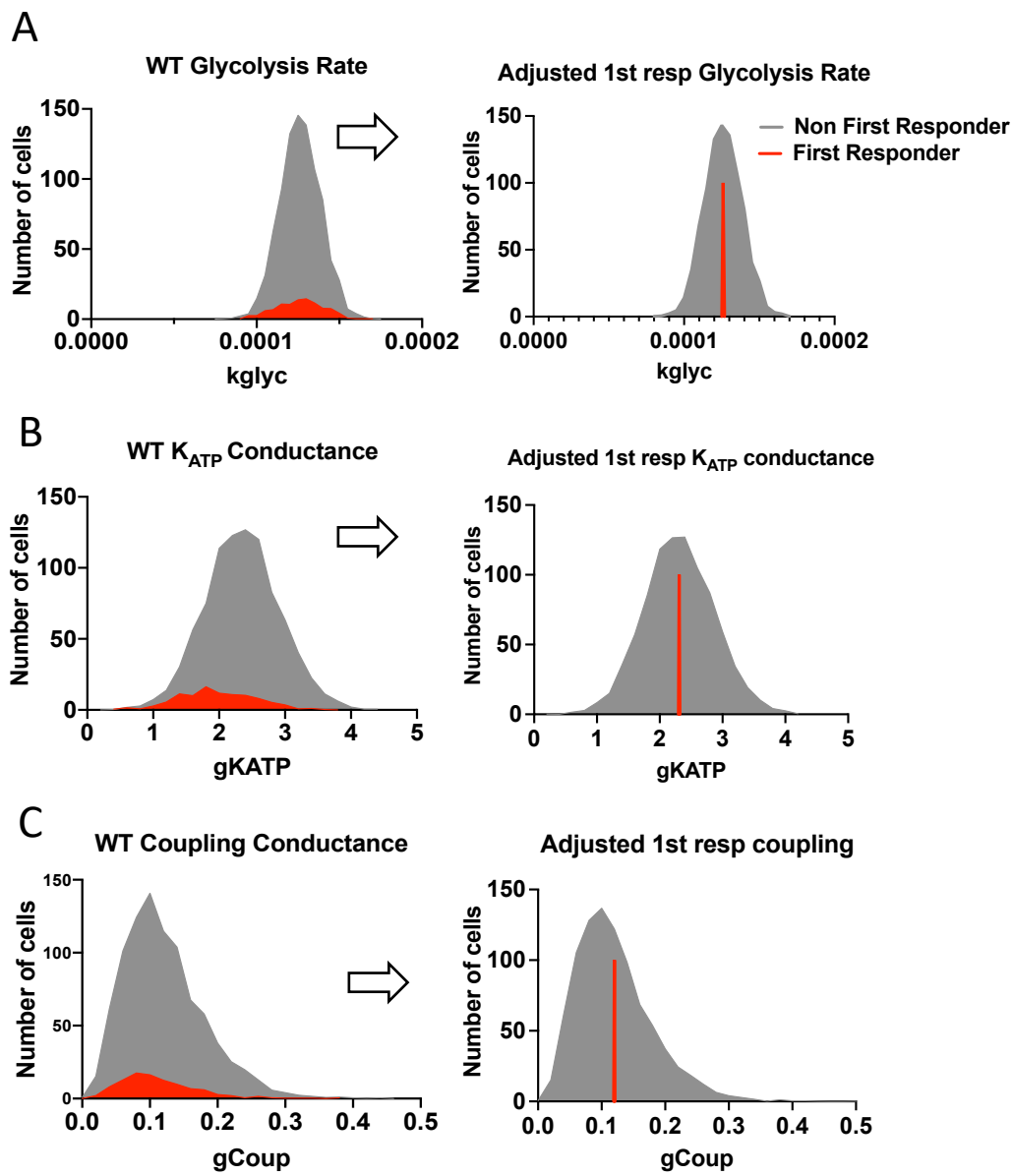

Figure S6

Supplement: S6 Fig — (A) Distribution of the glycolysis rate (kglyc) in non-first responders (gray) and first responders (red) before (left) and after (right) parameter adjustment. Adjustment was done to set first responder parameter to be the same as the islet-average value (n = 5 seeds). (B) As in (A) for adjustment of KATP conductance (gKATP). (C) As in (A) for adjustment of coupling conductance (gcoup). See S10 Data file for values used in each graph. (PDF) [file pbio.3001761.s006.pdf]

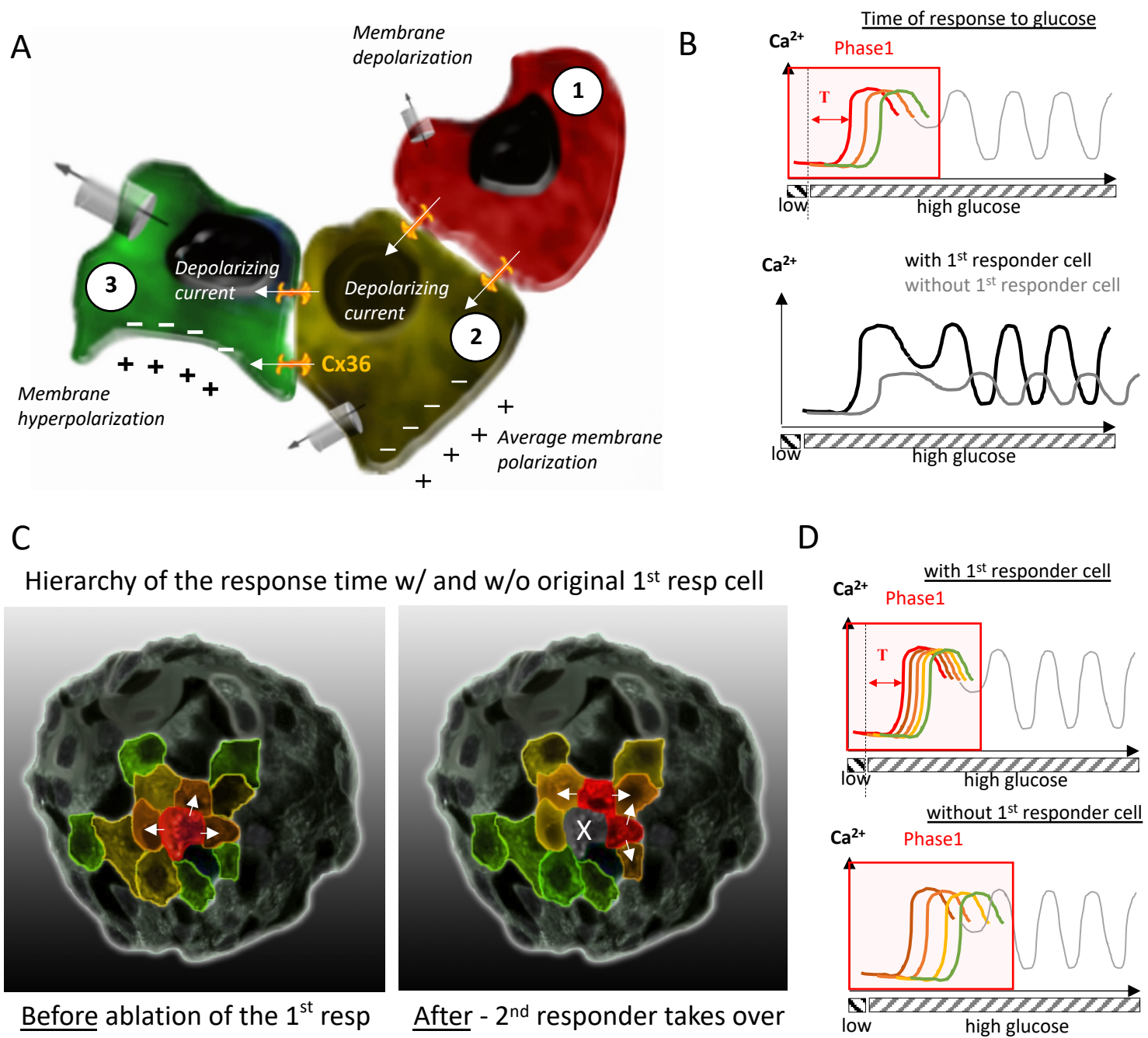

Figure S7

Supplement: S7 Fig — (A) Cell1—first responder cell (red) is more prone to membrane depolarization upon glucose stimulation. Cell 2 (yellow) is less depolarized than a first responder. The difference in the membrane potential between the cell 1 and 2 leads to depolarizing current to flow through the gap junctions into cell 2 triggering depolarization. Cell 2 subsequently depolarizes the less excitable cell 3 (green). (B) Representation of the [Ca2+] response to glucose in cells 1,2,3 shown in (A) and the islet-average [Ca2+] response with (black) and without (gray) the first responder cell. (C) Schematic of the time of response in the islet (red—faster response, green—slower) before and after the first responder ablation. Post-ablation, the cell with the second earliest response time takes over the role of the first responder. (D) Representation of the [Ca2+] coordination before and dis-coordination after the first responder cell ablation. (PDF) [file pbio.3001761.s007.pdf]
