# Supplementary material for: Functional architecture of pancreatic islets identifies a population of first responder cells that drive the first-phase calcium response
Source: PLoS Biol. 2022 Sep 13;20(9):e3001761. doi: 10.1371/journal.pbio.3001761 (PMC9506623; doi:10.1371/journal.pbio.3001761)
Supplement: S4 Fig — (A) Quantification of the Ca2+ oscillation period and (B) frequency during initial and repeated glucose elevation (n = 4 islets). No significant difference was found in 1 sample t test with initial-repeated period (frequency) difference compared to 0. (C) Response time of all cells in the optical section of the islet during initial vs. during repeated glucose elevation. Red dots represent first responder and green represent last responder cells identified during the initial glucose elevation. See S8 Data file for values used in each graph. (PDF) [file pbio.3001761.s004.pdf]

Response time ($T-T_{1st}$) of all cells in each islet during initial and repeated glucose elevation

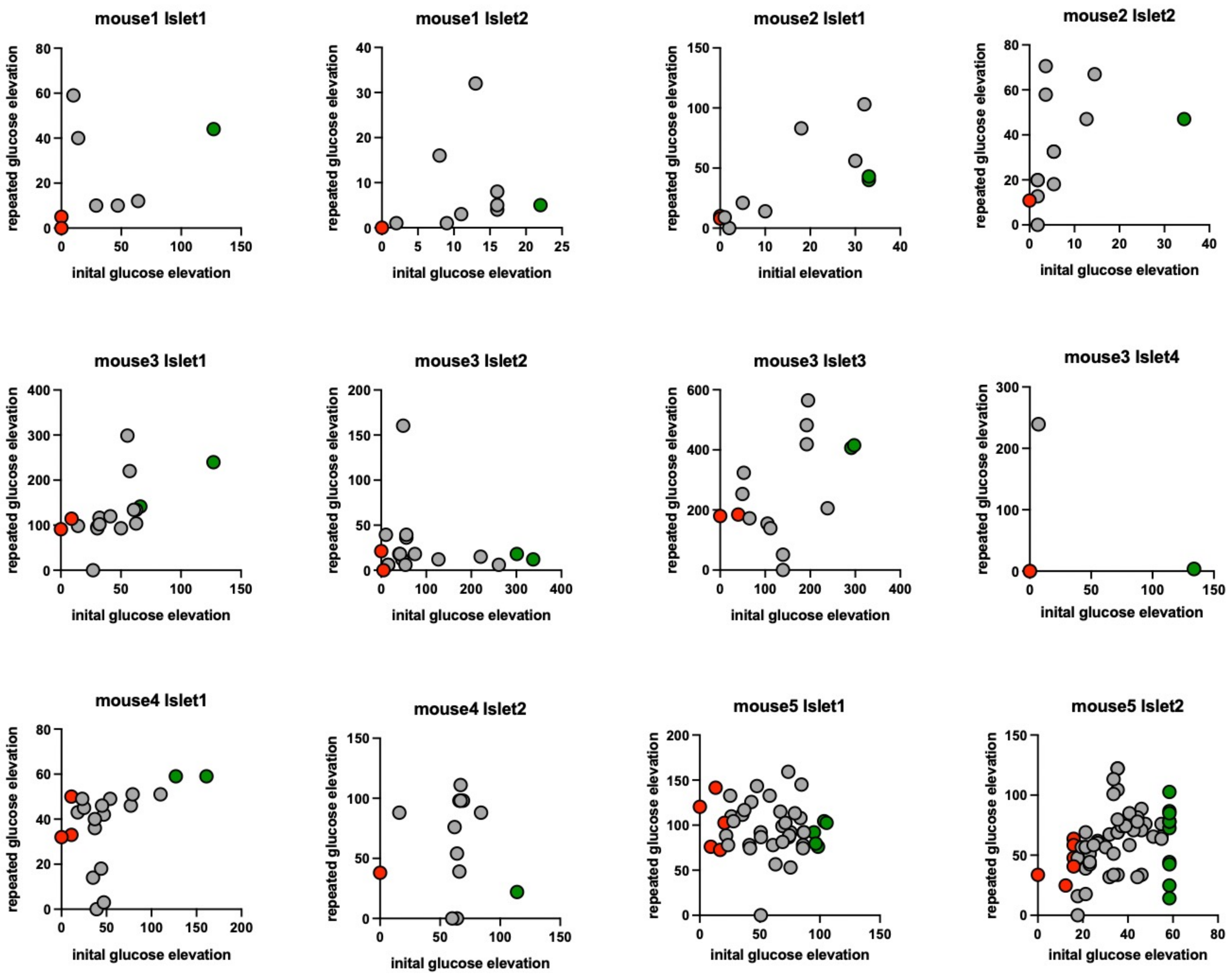

Figure S4
