# Supplementary material for: Functional architecture of pancreatic islets identifies a population of first responder cells that drive the first-phase calcium response
Source: PLoS Biol. 2022 Sep 13;20(9):e3001761. doi: 10.1371/journal.pbio.3001761 (PMC9506623; doi:10.1371/journal.pbio.3001761)
Supplement: S5 Fig — (A) Left pie chart: Percent of islets in which initial first and last responders remain in their role during repeated glucose elevation. Middle pie chart: same as left but following control cell ablation. Right pie charts: same as middle but following first responder cell ablation. (B) Islet size dependence of the [Ca2+] influx into the islet (area under the curve of the [Ca2+] time course) for random and first responder ablation cases. (C) Size distribution of the islets studied in the ablation experiments. See S9 Data file for values used in each graph. (PDF) [file pbio.3001761.s005.pdf]

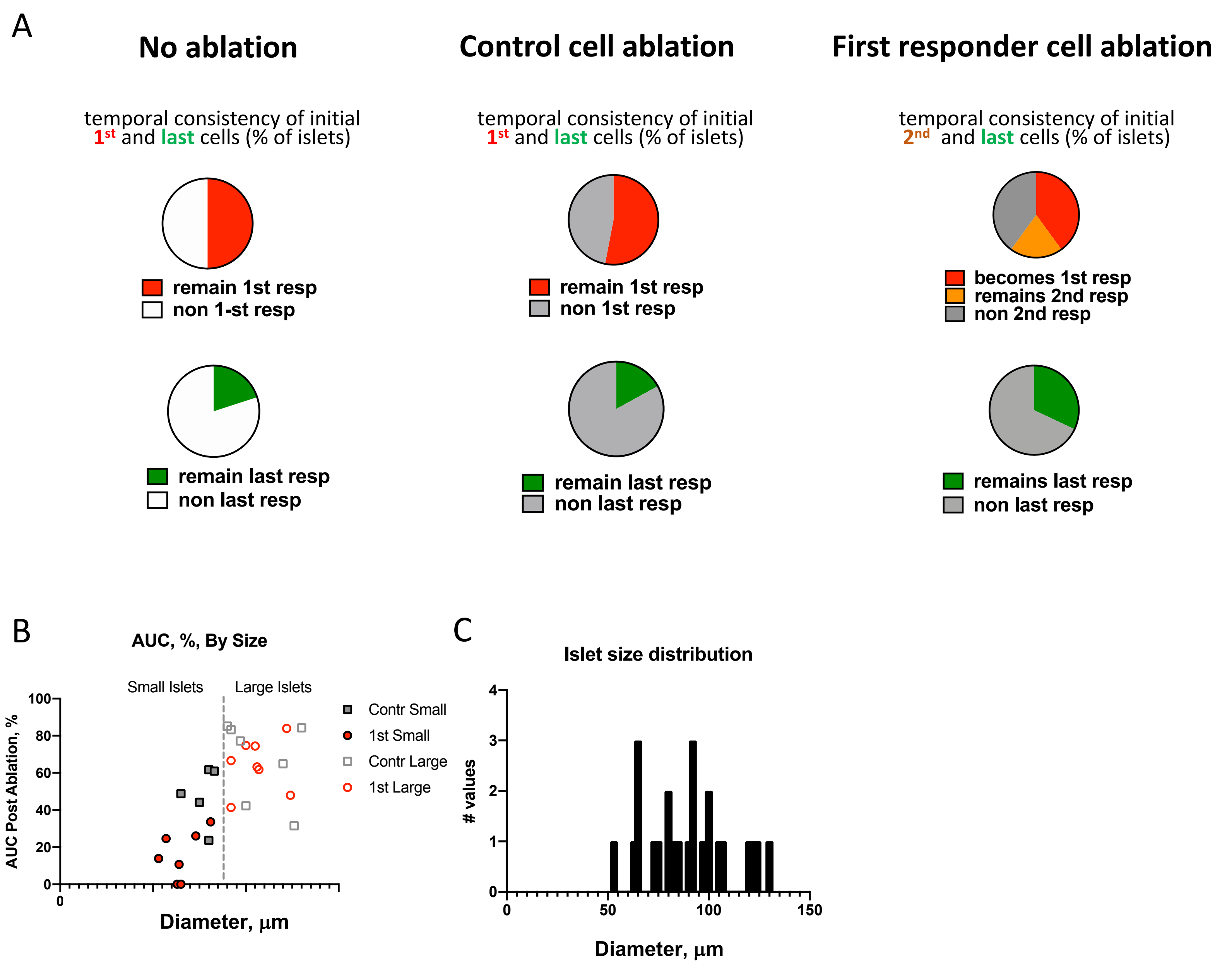
A
No ablation
Control cell ablation
First responder cell ablation
temporal consistency of initial
1st and last cells (% of islets)
remain 1st resp
non 1-st resp
remain last resp
non last resp
temporal consistency of initial
1st and last cells (% of islets)
remain 1st resp
non 1st resp
remain last resp
non last resp
temporal consistency of initial
2nd and last cells (% of islets)
becomes 1st resp
remains 2nd resp
non 2nd resp
remains last resp
non last resp
B
AUC, %, By Size
Small Islets
Large Islets
AUC Post Ablation, %
Diameter, µm
Contr Small
1st Small
Contr Large
1st Large
C
Islet size distribution
values
Diameter, µm

Figure S5
