# Supplementary material for: Functional architecture of pancreatic islets identifies a population of first responder cells that drive the first-phase calcium response
Source: PLoS Biol. 2022 Sep 13;20(9):e3001761. doi: 10.1371/journal.pbio.3001761 (PMC9506623; doi:10.1371/journal.pbio.3001761)
Supplement: S1 Table — (PDF) [file pbio.3001761.s008.pdf]

**Parameter**	**Description of parameter**	**Distribution**	**Mean $\pm$ Std Dev**	**units**
g_{KATP}	Max conductance of K_{ATP} channel current	Normal	2.31 $\pm$ 0.57	pA mV^{-1}
k_{glc}	Rate constant of glycolysis	Normal	0.000126 $\pm$ 0.0000315	ms^{-1}
g_{Coup}	Coupling conductance	Gamma, k=θ=4	120 $\pm$ 30	ps
g_{KTO}	Conductance of $I_{KCa(BK)}$ (voltage and Ca^{2+}) dependent transient outward K^+ current	Normal	2.13 $\pm$ 0.213	pA mV^{-1}
P_{SERCA}	Maximum rate of pumping Ca^{2+} into ER	Normal	0.096 $\pm$ 0.0096	amole ms^{-1}
P_{NaCa}	Maximum amplitude of I_{NaCa}, Na^+/Ca^{2+} exchanger	Normal	204 $\pm$ 20	pA
P_{rel}	Converting factor for Ca^{2+} release from ER	Normal	0.46 $\pm$ 0.46	fl ms^{-1}
P_{op}	Maximum rate of ATP production from oxphos	Normal	0.0005 $\pm$ 0.00005	ms^{-1}
$[ATP_{tot}]$	Total amount of ATP species	Normal	4 $\pm$ 0.4	mM

Supplemental Table
