# Supplementary material for: Functional architecture of pancreatic islets identifies a population of first responder cells that drive the first-phase calcium response
Source: PLoS Biol. 2022 Sep 13;20(9):e3001761. doi: 10.1371/journal.pbio.3001761 (PMC9506623; doi:10.1371/journal.pbio.3001761)
Supplement: S1 Statistical analysis LMEM — (DOCX) [file pbio.3001761.s019.docx]

**1 Figure 2K**

This is the overall summary of the model. The parameter labeled “phi” is the correlation between times within the same cell.

Linear mixed-effects model fit by REML

Data: fig2k_sum_long

AIC BIC logLik

443.9598 460.0175 -212.9799

Random effects:

Formula: ~1 | mouse

(Intercept)

StdDev: 0.001913688

Formula: ~1 | islet %in% mouse

(Intercept) Residual

StdDev: 13.25065 25.55243

Correlation Structure: AR(1)

Formula: ~1 | mouse/islet

Parameter estimate(s):

Phi

-0.2496282

Fixed effects: value ~ time

Value Std.Error DF t-value p-value

(Intercept) 5.99689 8.090846 38 0.7411948 0.4631

time6-12 19.86041 10.879465 38 1.8254948 0.0758

time18-24 27.06229 11.013295 38 2.4572387 0.0187

time30-36 44.41009 15.529664 38 2.8596944 0.0069

time42-48 34.21587 12.607091 38 2.7140175 0.0099

Correlation:

(Intr) tm6-12 t18-24 t30-36

time6-12 -0.535

time18-24 -0.564 0.412

time30-36 -0.367 0.368 0.243

time42-48 -0.383 0.237 0.333 0.186

Standardized Within-Group Residuals:

Min Q1 Med Q3 Max

-1.9018680 -0.6883790 -0.1980082 0.2904159 2.5107550

Number of Observations: 49

Number of Groups:

mouse islet %in% mouse

3 7

**1.1 ANOVA table**

The p-value for time is the overall test of the effect of time.

|  | **numDF** | **denDF** | **F-value** | **p-value** |
| --- | --- | --- | --- | --- |
| (Intercept) | 1 | 38 | 0.5493698 | 0.4631314 |
| time | 4 | 38 | 3.6403937 | 0.0131832 |

**1.2 Estimated means**

The table below provides estimates of each mean.

| **time** | **emmean** | **SE** | **df** | **lower.CL** | **upper.CL** |
| --- | --- | --- | --- | --- | --- |
| 0 | 5.996893 | 8.090846 | 2 | -28.815207 | 40.80899 |
| 6-12 | 25.857301 | 9.469101 | 2 | -14.884954 | 66.59956 |
| 18-24 | 33.059188 | 9.283398 | 2 | -6.884048 | 73.00242 |
| 30-36 | 50.406987 | 14.645903 | 2 | -12.609249 | 113.42322 |
| 42-48 | 40.212760 | 12.090760 | 2 | -11.809581 | 92.23510 |

**1.3 Pairwise comparison of means with Tukey’s HSD adjustment for multiple testing**

The table below provides a comparison of each pairwise combination of means.

| **contrast** | **estimate** | **SE** | **df** | **t.ratio** | **p.value** |
| --- | --- | --- | --- | --- | --- |
| 0 - (6-12) | -19.860407 | 10.87947 | 38 | -1.8254948 | 0.3744063 |
| 0 - (18-24) | -27.062295 | 11.01330 | 38 | -2.4572387 | 0.1223213 |
| 0 - (30-36) | -44.410093 | 15.52966 | 38 | -2.8596944 | 0.0503963 |
| 0 - (42-48) | -34.215866 | 12.60709 | 38 | -2.7140175 | 0.0704089 |
| (6-12) - (18-24) | -7.201887 | 11.87218 | 38 | -0.6066189 | 0.9731879 |
| (6-12) - (30-36) | -24.549686 | 15.33710 | 38 | -1.6006728 | 0.5061465 |
| (6-12) - (42-48) | -14.355459 | 14.56640 | 38 | -0.9855187 | 0.8601436 |
| (18-24) - (30-36) | -17.347799 | 16.71792 | 38 | -1.0376768 | 0.8362710 |
| (18-24) - (42-48) | -7.153572 | 13.70478 | 38 | -0.5219762 | 0.9845854 |
| (30-36) - (42-48) | 10.194227 | 18.09159 | 38 | 0.5634787 | 0.9795323 |

**1.4 Comparison of each mean to fixed value islet median=50**

| **contrast** | **estimate** | **SE** | **df** | **t.ratio** | **p.value** |
| --- | --- | --- | --- | --- | --- |
| m0 | -44.0031068 | 8.090846 | 2 | -5.4386287 | 0.0321849 |
| m6_12 | -24.1426995 | 9.469101 | 2 | -2.5496294 | 0.1255157 |
| m18_24 | -16.9408121 | 9.283398 | 2 | -1.8248504 | 0.2095754 |
| m30_36 | 0.4069865 | 14.645903 | 2 | 0.0277884 | 0.9803544 |
| m42_48 | -9.7872404 | 12.090760 | 2 | -0.8094810 | 0.5032325 |

**2 Figure 2L**

This is the overall summary of the model. The parameter labeled “phi” is the correlation between times within the same cell.

Linear mixed-effects model fit by REML

Data: fig2L_sum_long

AIC BIC logLik

379.2248 394.4247 -180.6124

Random effects:

Formula: ~1 | mouse

(Intercept)

StdDev: 0.001162736

Formula: ~1 | islet %in% mouse

(Intercept) Residual

StdDev: 6.64031e-05 19.98899

Correlation Structure: AR(1)

Formula: ~1 | mouse/islet

Parameter estimate(s):

Phi

0.2522794

Fixed effects: value ~ time

Value Std.Error DF t-value p-value

(Intercept) 96.07641 5.178941 34 18.551360 0.0000

time6-12 -21.57147 7.077477 34 -3.047904 0.0044

time18-24 -24.85470 6.742433 34 -3.686310 0.0008

time30-36 -55.61240 11.894994 34 -4.675278 0.0000

time42-48 -38.19451 9.782290 34 -3.904455 0.0004

Correlation:

(Intr) tm6-12 t18-24 t30-36

time6-12 -0.568

time18-24 -0.526 0.381

time30-36 -0.366 0.235 0.267

time42-48 -0.461 0.363 0.291 0.327

Standardized Within-Group Residuals:

Min Q1 Med Q3 Max

-2.09223669 -0.39546461 -0.09887468 0.33493736 2.10706481

Number of Observations: 45

Number of Groups:

mouse islet %in% mouse

3 7

**2.1 ANOVA table**

The p-value for time is the overall test of the effect of time.

|  | **numDF** | **denDF** | **F-value** | **p-value** |
| --- | --- | --- | --- | --- |
| (Intercept) | 1 | 34 | 344.15295 | 0.00e+00 |
| time | 4 | 34 | 8.19892 | 9.66e-05 |

**2.2 Estimated means**

The table below provides estimates of each mean.

| **time** | **emmean** | **SE** | **df** | **lower.CL** | **upper.CL** |
| --- | --- | --- | --- | --- | --- |
| 0 | 96.07641 | 5.178941 | 2 | 73.793219 | 118.35959 |
| 6-12 | 74.50494 | 5.939102 | 2 | 48.951045 | 100.05883 |
| 18-24 | 71.22170 | 5.964934 | 2 | 45.556665 | 96.88674 |
| 30-36 | 40.46401 | 11.101010 | 2 | -7.299786 | 88.22780 |
| 42-48 | 57.88190 | 8.706275 | 2 | 20.421817 | 95.34198 |

**2.3 Pairwise comparison of means with Tukey’s HSD adjustment for multiple testing**

The table below provides a comparison of each pairwise combination of means.

| **contrast** | **estimate** | **SE** | **df** | **t.ratio** | **p.value** |
| --- | --- | --- | --- | --- | --- |
| 0 - (6-12) | 21.571466 | 7.077477 | 34 | 3.0479035 | 0.0336467 |
| 0 - (18-24) | 24.854701 | 6.742433 | 34 | 3.6863102 | 0.0066074 |
| 0 - (30-36) | 55.612398 | 11.894994 | 34 | 4.6752775 | 0.0004104 |
| 0 - (42-48) | 38.194509 | 9.782290 | 34 | 3.9044547 | 0.0036551 |
| (6-12) - (18-24) | 3.283235 | 7.694331 | 34 | 0.4267083 | 0.9927604 |
| (6-12) - (30-36) | 34.040932 | 12.329212 | 34 | 2.7609982 | 0.0654215 |
| (6-12) - (42-48) | 16.623043 | 9.769998 | 34 | 1.7014378 | 0.4465767 |
| (18-24) - (30-36) | 30.757697 | 12.007820 | 34 | 2.5614723 | 0.1007382 |
| (18-24) - (42-48) | 13.339809 | 10.139319 | 34 | 1.3156513 | 0.6835560 |
| (30-36) - (42-48) | -17.417889 | 12.695335 | 34 | -1.3719912 | 0.6491562 |

**2.4 Comparison of each mean to fixed value islet median=50**

| **contrast** | **estimate** | **SE** | **df** | **t.ratio** | **p.value** |
| --- | --- | --- | --- | --- | --- |
| m0 | 46.076405 | 5.178941 | 2 | 8.8968772 | 0.0123991 |
| m6_12 | 24.504939 | 5.939102 | 2 | 4.1260343 | 0.0540240 |
| m18_24 | 21.221705 | 5.964934 | 2 | 3.5577434 | 0.0707253 |
| m30_36 | -9.535993 | 11.101010 | 2 | -0.8590202 | 0.4808494 |
| m42_48 | 7.881896 | 8.706275 | 2 | 0.9053121 | 0.4608553 |
