# Supplementary material for: Functional architecture of pancreatic islets identifies a population of first responder cells that drive the first-phase calcium response
Source: PLoS Biol. 2022 Sep 13;20(9):e3001761. doi: 10.1371/journal.pbio.3001761 (PMC9506623; doi:10.1371/journal.pbio.3001761)
Supplement: S2 Statistical analysis LMEM — (DOCX) [file pbio.3001761.s020.docx]

**Vira Kravets - Mixed model - updates for PLOS BIO**

**Laura Pyle**

**03 May 2022**

**1 Figure 1f**

The parameter labeled “rho” is the correlation between measures on the same mouse or islet. Because the sample size is relatively small, I needed to use a simple correlation structure with the same correlation between measures within a mouse as between measures within an islet. The model still accounts for both levels of correlation.

Linear mixed-effects model fit by REML

Data: fig1f_long

AIC BIC logLik

-39.35122 0.7874933 30.67561

Random effects:

Formula: ~1 | mouse

(Intercept)

StdDev: 9.738517e-08

Formula: ~1 | islet %in% mouse

(Intercept) Residual

StdDev: 1.942382e-06 0.2085432

Correlation Structure: Compound symmetry

Formula: ~1 | mouse/islet

Parameter estimate(s):

Rho

-0.02272727

Fixed effects: value ~ Characteristic

Value Std.Error DF t-value p-value

(Intercept) 0.9278185 0.03526795 271 26.307693 0.0000

Characteristichub.like..p2. 0.0786085 0.04979323 271 1.578699 0.1156

CharacteristicIslet 0.0801068 0.04563860 271 1.755241 0.0803

Characteristiclast.resp 0.3272542 0.04938029 271 6.627224 0.0000

Characteristicw.end 0.0839583 0.05212051 271 1.610850 0.1084

Characteristicw.or 0.0356345 0.05321780 271 0.669598 0.5037

CharacteristicX1st.resp -0.0671516 0.04717662 271 -1.423409 0.1558

Correlation:

(Intr) C...2. ChrctI Chrctrstcl. Chrctrstcw.n

Characteristichub.like..p2. -0.878

CharacteristicIslet -0.878 0.648

Characteristiclast.resp -0.766 0.641 0.634

Characteristicw.end -0.776 0.564 0.611 0.561

Characteristicw.or -0.735 0.570 0.592 0.531 0.512

CharacteristicX1st.resp -0.809 0.679 0.664 0.580 0.591

Chrctrstcw.r

Characteristichub.like..p2.

CharacteristicIslet

Characteristiclast.resp

Characteristicw.end

Characteristicw.or

CharacteristicX1st.resp 0.558

Standardized Within-Group Residuals:

Min Q1 Med Q3 Max

-3.07663370 -0.28731503 -0.03800277 0.29621993 5.90605837

Number of Observations: 291

Number of Groups:

mouse islet %in% mouse

8 14

## 1.1 ANOVA table

The p-value for Characteristic is the overall test of the effect.

|  | **numDF** | **denDF** | **F-value** | **p-value** |
| --- | --- | --- | --- | --- |
| (Intercept) | 1 | 271 | 692.09472 | 0 |
| Characteristic | 6 | 271 | 14.48708 | 0 |

**1.2 Estimated means**

The table below provides estimates of each mean.

| **Characteristic** | **emmean** | **SE** | **df** | **lower.CL** | **upper.CL** |
| --- | --- | --- | --- | --- | --- |
| hub.like..p1. | 0.9278185 | 0.0352680 | 7 | 0.8444230 | 1.0112139 |
| hub.like..p2. | 1.0064270 | 0.0252698 | 7 | 0.9466735 | 1.0661805 |
| Islet | 1.0079252 | 0.0223772 | 7 | 0.9550115 | 1.0608389 |
| last.resp | 1.2550727 | 0.0318616 | 7 | 1.1797319 | 1.3304135 |
| w.end | 1.0117768 | 0.0332541 | 7 | 0.9331433 | 1.0904103 |
| w.or | 0.9634530 | 0.0362905 | 7 | 0.8776396 | 1.0492664 |
| X1st.resp | 0.8606668 | 0.0279027 | 7 | 0.7946875 | 0.9266461 |

**1.3 Pairwise comparison of means with Tukey’s HSD adjustment for multiple testing**

The table below provides a comparison of each pairwise combination of means.

| **contrast** | **estimate** | **SE** | **df** | **t.ratio** | **p.value** |
| --- | --- | --- | --- | --- | --- |
| hub.like..p1. - hub.like..p2. | -0.0786085 | 0.0497932 | 271 | -1.5786987 | 0.6960917 |
| hub.like..p1. - Islet | -0.0801068 | 0.0456386 | 271 | -1.7552414 | 0.5794753 |
| hub.like..p1. - last.resp | -0.3272542 | 0.0493803 | 271 | -6.6272244 | 0.0000000 |
| hub.like..p1. - w.end | -0.0839583 | 0.0521205 | 271 | -1.6108497 | 0.6754697 |
| hub.like..p1. - w.or | -0.0356345 | 0.0532178 | 271 | -0.6695983 | 0.9941156 |
| hub.like..p1. - X1st.resp | 0.0671516 | 0.0471766 | 271 | 1.4234095 | 0.7888078 |
| hub.like..p2. - Islet | -0.0014982 | 0.0402238 | 271 | -0.0372478 | 1.0000000 |
| hub.like..p2. - last.resp | -0.2486457 | 0.0420372 | 271 | -5.9148906 | 0.0000002 |
| hub.like..p2. - w.end | -0.0053498 | 0.0476037 | 271 | -0.1123819 | 0.9999998 |
| hub.like..p2. - w.or | 0.0429740 | 0.0478556 | 271 | 0.8979918 | 0.9726497 |
| hub.like..p2. - X1st.resp | 0.1457602 | 0.0389259 | 271 | 3.7445588 | 0.0041037 |
| Islet - last.resp | -0.2471475 | 0.0407684 | 271 | -6.0622265 | 0.0000001 |
| Islet - w.end | -0.0038515 | 0.0435035 | 271 | -0.0885343 | 1.0000000 |
| Islet - w.or | 0.0444722 | 0.0451451 | 271 | 0.9850956 | 0.9569061 |
| Islet - X1st.resp | 0.1472584 | 0.0380877 | 271 | 3.8662962 | 0.0026185 |
| last.resp - w.end | 0.2432959 | 0.0476220 | 271 | 5.1088955 | 0.0000126 |
| last.resp - w.or | 0.2916197 | 0.0498184 | 271 | 5.8536553 | 0.0000003 |
| last.resp - X1st.resp | 0.3944059 | 0.0443119 | 271 | 8.9006726 | 0.0000000 |
| w.end - w.or | 0.0483238 | 0.0520489 | 271 | 0.9284307 | 0.9677207 |
| w.end - X1st.resp | 0.1511099 | 0.0451004 | 271 | 3.3505205 | 0.0158765 |
| w.or - X1st.resp | 0.1027862 | 0.0474857 | 271 | 2.1645736 | 0.3184164 |

**2 Figure 1g**

This is the overall summary of the model. However, most of the information you will need is below in the ANOVA table, estimated means, and pairwise comparisons.

The parameter labeled “rho” is the correlation between measures on the same mouse or islet. Because the sample size is relatively small, I needed to use a simple correlation structure with the same correlation between measures within a mouse as between measures within an islet. The model still accounts for both levels of correlation.

Linear mixed-effects model fit by REML

Data: fig1g_long

AIC BIC logLik

1254.601 1290.716 -616.3005

Random effects:

Formula: ~1 | mouse

(Intercept)

StdDev: 0.0001385423

Formula: ~1 | islet %in% mouse

(Intercept) Residual

StdDev: 0.0001189538 5.2232

Correlation Structure: Compound symmetry

Formula: ~1 | mouse/islet

Parameter estimate(s):

Rho

-0.02325581

Fixed effects: value ~ Characteristic

Value Std.Error DF t-value p-value

(Intercept) 0.702989 1.129752 186 0.622251 0.5345

Characteristichub.like..p2. -0.487730 1.664667 186 -0.292989 0.7699

CharacteristicIslet -0.602662 1.443451 186 -0.417515 0.6768

Characteristiclast.resp 0.400227 1.472905 186 0.271726 0.7861

Characteristicw.end 6.712163 1.549431 186 4.332017 0.0000

Characteristicw.or -9.801586 1.560092 186 -6.282697 0.0000

CharacteristicX1st.resp -3.614844 1.485456 186 -2.433491 0.0159

Correlation:

(Intr) C...2. ChrctI Chrctrstcl. Chrctrstcw.n

Characteristichub.like..p2. -0.887

CharacteristicIslet -0.879 0.639

Characteristiclast.resp -0.805 0.685 0.679

Characteristicw.end -0.806 0.610 0.654 0.623

Characteristicw.or -0.780 0.626 0.645 0.603 0.591

CharacteristicX1st.resp -0.804 0.683 0.677 0.620 0.621

Chrctrstcw.r

Characteristichub.like..p2.

CharacteristicIslet

Characteristiclast.resp

Characteristicw.end

Characteristicw.or

CharacteristicX1st.resp 0.602

Standardized Within-Group Residuals:

Min Q1 Med Q3 Max

-4.5763545 -0.2967332 -0.0192080 0.3754480 6.1465857

Number of Observations: 204

Number of Groups:

mouse islet %in% mouse

6 12

**2.1 ANOVA table**

The p-value for Characteristic is the overall test of the effect.

|  | **numDF** | **denDF** | **F-value** | **p-value** |
| --- | --- | --- | --- | --- |
| (Intercept) | 1 | 186 | 0.3871962 | 0.5345389 |
| Characteristic | 6 | 186 | 25.4444370 | 0.0000000 |

**2.2 Estimated means**

The table below provides estimates of each mean.

| **Characteristic** | **emmean** | **SE** | **df** | **lower.CL** | **upper.CL** |
| --- | --- | --- | --- | --- | --- |
| hub.like..p1. | 0.7029890 | 1.1297516 | 5 | -2.201130 | 3.6071080 |
| hub.like..p2. | 0.2152593 | 0.8424520 | 5 | -1.950332 | 2.3808511 |
| Islet | 0.1003272 | 0.7019241 | 5 | -1.704026 | 1.9046805 |
| last.resp | 1.1032161 | 0.8747080 | 5 | -1.145292 | 3.3517245 |
| w.end | 7.4151520 | 0.9254023 | 5 | 5.036330 | 9.7939745 |
| w.or | -9.0985969 | 0.9801132 | 5 | -11.618058 | -6.5791358 |
| X1st.resp | -2.9118547 | 0.8850185 | 5 | -5.186867 | -0.6368422 |

**2.3 Pairwise comparison of means with Tukey’s HSD adjustment for multiple testing**

The table below provides a comparison of each pairwise combination of means.

| **contrast** | **estimate** | **SE** | **df** | **t.ratio** | **p.value** |
| --- | --- | --- | --- | --- | --- |
| hub.like..p1. - hub.like..p2. | 0.4877297 | 1.664667 | 186 | 0.2929893 | 0.9999473 |
| hub.like..p1. - Islet | 0.6026618 | 1.443451 | 186 | 0.4175146 | 0.9995840 |
| hub.like..p1. - last.resp | -0.4002271 | 1.472905 | 186 | -0.2717263 | 0.9999662 |
| hub.like..p1. - lagger | -6.7121630 | 1.549431 | 186 | -4.3320174 | 0.0004760 |
| hub.like..p1. - leader | 9.8015859 | 1.560092 | 186 | 6.2826970 | 0.0000000 |
| hub.like..p1. - 1st.resp | 3.6148436 | 1.485456 | 186 | 2.4334907 | 0.1906781 |
| hub.like..p2. - Islet | 0.1149322 | 1.335013 | 186 | 0.0860907 | 1.0000000 |
| hub.like..p2. - last.resp | -0.8879568 | 1.258296 | 186 | -0.7056818 | 0.9921322 |
| hub.like..p2. - lagger | -7.1998927 | 1.422784 | 186 | -5.0604243 | 0.0000205 |
| hub.like..p2. - leader | 9.3138563 | 1.397620 | 186 | 6.6640847 | 0.0000000 |
| hub.like..p2. - 1st.resp | 3.1271140 | 1.264603 | 186 | 2.4728037 | 0.1753625 |
| Islet - last.resp | -1.0028890 | 1.169578 | 186 | -0.8574792 | 0.9782052 |
| Islet - lagger | -7.3148248 | 1.249180 | 186 | -5.8557002 | 0.0000004 |
| Islet - leader | 9.1989241 | 1.270591 | 186 | 7.2398803 | 0.0000000 |
| Islet - 1st.resp | 3.0121818 | 1.177423 | 186 | 2.5582833 | 0.1451725 |
| last.resp - lagger | -6.3119359 | 1.314735 | 186 | -4.8009201 | 0.0000657 |
| last.resp - leader | 10.2018130 | 1.354239 | 186 | 7.5332427 | 0.0000000 |
| last.resp - 1st.resp | 4.0150708 | 1.289489 | 186 | 3.1136902 | 0.0342739 |
| lagger - leader | 16.5137489 | 1.406864 | 186 | 11.7379840 | 0.0000000 |
| lagger - 1st.resp | 10.3270067 | 1.323033 | 186 | 7.8055529 | 0.0000000 |
| leader - 1st.resp | -6.1867423 | 1.360378 | 186 | -4.5478104 | 0.0001953 |
