# Supplementary material for: Functional architecture of pancreatic islets identifies a population of first responder cells that drive the first-phase calcium response
Source: PLoS Biol. 2022 Sep 13;20(9):e3001761. doi: 10.1371/journal.pbio.3001761 (PMC9506623; doi:10.1371/journal.pbio.3001761)
Supplement: S3 Statistical analysis LMEM — (DOCX) [file pbio.3001761.s021.docx]

# Figure S2

The model would not converge with a correlation structure of cell within islet within mouse, so a simpler correlation structure was used: cell within islet. This was compared to a model with a correlation structure of cell within mouse, but the first model had a better fit to the data. The parameter labeled “phi” is the estimate of the correlation.

This is the overall summary of the model. The slope of the regression of response time on distance can be found in the table labeled “fixed effects,” under the column labeled “Value,” in the row labeled “Distance_1st_resp.”

Linear mixed-effects model fit by REML

Data: figs2

AIC BIC logLik

2407.101 2435.532 -1196.551

Random effects:

Formula: ~1 + Response_time | unique_islet

Structure: General positive-definite, Log-Cholesky parametrization

StdDev Corr

(Intercept) 0.004264545 (Intr)

Response_time 0.013286320 -0.014

Residual 39.381753025

Correlation Structure: AR(1)

Formula: ~1 | unique_islet

Parameter estimate(s):

Phi

0.9959679

Fixed effects: Distance_1st_resp ~ Response_time

Value Std.Error DF t-value p-value

(Intercept) 35.06951 9.601133 414 3.652643 0.0003

Response_time 0.02068 0.007111 414 2.908040 0.0038

Correlation:

(Intr)

Response_time -0.023

Standardized Within-Group Residuals:

Min Q1 Med Q3 Max

-0.9245278 -0.2733919 0.1109971 0.5370418 2.1964949

Number of Observations: 431

Number of Groups: 16

## ANOVA table

The p-value for Distance_1st_resp is the test of whether there is a significant non-zero slope.

|  | **numDF** | **denDF** | **F-value** | **p-value** |
| --- | --- | --- | --- | --- |
| (Intercept) | 1 | 414 | 13.341802 | 0.0002929 |
| Response_time | 1 | 414 | 8.456699 | 0.0038328 |

## Individual slopes

These are the estimates of the islet-level intercepts and slopes.

|  | **(Intercept)** | **Response_time** |
| --- | --- | --- |
| mouse1_Isl1 (d=112um) | 35.06954 | 0.0150783 |
| mouse1_Isl2 (d=88um) | 35.06952 | 0.0194637 |
| mouse1_Isl3 (d=95um) | 35.06951 | 0.0213184 |
| mouse2_Isl1 (d=109um) | 35.06951 | 0.0220071 |
| mouse3_Isl1 (d=80um) | 35.06951 | 0.0220864 |
| mouse4_Isl1 (d=71um) | 35.06948 | 0.0285712 |
| mouse5_Isl1 (d=68um) | 35.06951 | 0.0209504 |
| mouse6_Isl1 (d=66um) | 35.06951 | 0.0207016 |
| mouse6_Isl2 (d=61um) | 35.06950 | 0.0228136 |
| mouse7_Isl1 (d=135 um) | 35.06950 | 0.0238015 |
| mouse7_Isl2 (d=122um) | 35.06951 | 0.0214344 |
| mouse8_Isl2 (d=98um) | 35.06950 | 0.0227907 |
| mouse8_Isl5 (d=70um) 1_22_20 | 35.06960 | 0.0014067 |
| mouse8_Isl8 (d=77um) 1_22_20 | 35.06952 | 0.0195525 |
| mouse9_Isl1 (d=xum) 2_11_20 | 35.06951 | 0.0217412 |
| mouse9_Isl2 (d=xum) 2_11_20 | 35.06948 | 0.0271660 |
